# Research on the impact of traditional Chinese medicine registration innovation-oriented policies and enterprises performance: an empirical analysis based on listed enterprises in China

**DOI:** 10.3389/fpubh.2025.1666517

**Published:** 2025-11-26

**Authors:** Junfeng Lv, Kaidi Lu, Ming Xie, Wanping Sun, Yiming Liu

**Affiliations:** 1 The Second People’s Hospital of Huizhou, Huizhou, Guangdong, China; 2 Liaoning University of Traditional Chinese Medicine, Dalian, China

**Keywords:** medicine, Chinese traditional, herbal medicine, phytotherapy, policy making, health care reform

## Abstract

**Objective:**

The performance of traditional Chinese medicine (TCM) enterprises is affected by various factors, among which the impact of TCM Registration Innovation-Oriented policies (TCMRIPs) may be involved. TCMRIPs, in turn, improve enterprises performance by influencing TCM enterprises’ R&D investment. This aim of this study is to explore the impact of TCMRIPs on the performance of TCM enterprises, and the mediating role of R&D investment in this process.

**Methods:**

This study constructs an unbalanced panel fixed model to empirically analyze the impact of TCMRIPs on enterprise performance and their heterogeneous characteristics, and examines the mediating effect of R&D investment.

**Results:**

The results show that the performance of TCM listed enterprises in China has a significant positive correlation with TCMRIPs. TCMRIPs play a positive role in TCM listed enterprises performance.

**Conclusion:**

This study also indicates that R&D investment plays a mediating role in the effect of TCMRIPs on the performance of TCM listed enterprises in China, and the impact of policies on enterprise performance has a lag. The new version of the *Drug Registration Administration Measures*, the *TCM registration classification*, and the mechanisms they bring can promote the improvement of enterprise performance more effectively than the previous policies and mechanisms.

## 1 Introduction

Traditional Chinese medicine (TCM), as a culmination of China’s millennia-old medical wisdom (1), transcends mere medical practice; it represents a cultural treasure and a strategic asset that supports the nation’s autonomy in health and medical security (2). Its holistic approach to health, “emphasizing the balance of the human body and harmony with nature,” provides distinctive solutions to contemporary health challenges, including chronic disease management and preventive healthcare (3). In an age where global health security is increasingly linked to national sovereignty, TCM’s significance in reducing dependence on imported pharmaceuticals and ensuring domestic medical self-sufficiency has become more critical than ever (4, 5).

Drug registration and evaluation, serving as a fundamental gateway to access the pharmaceutical market, is a cornerstone of public health governance (6). This process is intricately tied to the health and well-being of Chinese citizens, ensuring the safety, efficacy, and quality of medicinal products (7). Additionally, it imposes strict regulations on the research and development, testing, and production practices of pharmaceutical companies (8). A robust registration system not only promotes innovation but also protects public health, fostering a win-win situation for both enterprises and society (9). Numerous countries have implemented standardized legal frameworks for drug registration and evaluation, underscoring a global acknowledgment of their significance (10). In the United States, for example, the Food and Drug Administration enforces stringent clinical trial requirements (11). The European Union depends on the European Medicines Agency to ensure the harmonization of standards among its member states (12, 13). Similarly, China has established a comprehensive set of drug registration regulations to oversee the research and development, testing, and production activities of domestic pharmaceutical companies, adapting in tandem with its expanding pharmaceutical sector and evolving public health demands, and are supervised and managed by the National Medical Products Administration (14).

Since its establishment, China’s drug registration and evaluation system has undergone several significant reforms, each marking an important step toward increased standardization and efficiency (15). The *Drug Administration Law*, officially enacted in 1985, established the initial legal framework for drug management in China. In 2001, the Chinese government revised this law to address new challenges in the pharmaceutical market (4). This revision strengthened regulatory oversight and increased penalties for non-compliance (16). In 2002, a significant milestone was reached when China updated its drug registration policy from the previous *New Drug Approval Measures* to the *Drug Registration Administration Measures*. This change streamlined the approval process and introduced more science-based evaluation criteria (17). Since then, this policy has been the guiding document for drug registration in China, with subsequent revisions reflecting advancements in pharmaceutical science and regulatory practices. The current drug registration process follows the 2020 version *Drug Registration Measures*. This version incorporates lessons learned from previous reforms and aligns more closely with international best practices (18). The inheritance, innovation, and development of TCM are based on these guiding documents and are specifically designed to address the unique characteristics of TCM (19). These Policies may contain many development strategies, among which there are also contents related to innovation (20).

In this study, we uniformly refer to the innovative-oriented TCM registration policies as TCMRIPs. These TCMRIPs have a significant impact not only on the performance of TCM enterprises but also on the overall trajectory of the TCM industry. While existing studies have demonstrated that innovation can greatly enhance enterprise performance, TCM possesses unique characteristics that differentiate it from Western pharmaceuticals and other industries, making general conclusions less applicable. Firstly, regarding material properties, TCM is far more complex than products in other industries, and it also differs from other drugs in the pharmaceutical industry (21). Most TCM preparations consist of compound formulas containing various Chinese medicinal materials, each of which contains 100 of chemical components (22). These components work together synergistically to create comprehensive therapeutic effects, making it challenging to define their mechanisms of action as precisely as those of chemical drugs, which typically contain a single active ingredient (23). Additionally, the quality of Chinese medicinal materials is significantly influenced by natural factors, including geographical origin, climate, and cultivation methods (24). This concept is reflected in the term “Daodi medicinal materials,” which highlights a distinct characteristic from other industries, where product quality can be controlled through standardized production processes (25). Secondly, the production process within the TCM industry is distinctive (26). In the upstream phase, the cultivation of Chinese medicinal materials relies heavily on natural conditions, features a lengthy growth cycle, and necessitates strict adherence to traditional harvesting seasons and processing methods that have been honed over centuries (27). In the midstream phase, the processing stage known as “Paozhi” employs unique techniques, as various processing methods can modify the medicinal properties of the materials to enhance their efficacy or reduce toxicity (28). This is not present in the production and processing of other industries. In clinical practice, the focus is on personalized diagnosis and treatment based on the principle of “syndrome differentiation.” This approach involves adjusting prescriptions according to each patient’s unique constitution and condition (29). This method significantly contrasts with the standardized application of chemical drugs, which typically follows the “one drug for one disease” model. Thirdly, regarding value evaluation systems, TCM extends beyond mere disease treatment; it encompasses prevention, healthcare, and rehabilitation, resonating with the global trend toward holistic health (30). Its value assessment often requires integration with long-term clinical practice and the distillation of experience, as many of its benefits emerge gradually over time. In contrast, the value of products in other industries is typically gauged by short-term efficacy, performance, and other quantifiable metrics, which lack the comprehensive and long-term perspective characteristic of TCM (31). The unique characteristics of TCM suggest that empirical analyses focusing on industrial manufacturing or the pharmaceutical sector may not apply to TCM enterprises (32). The complexity of TCM’s material foundation, the distinctiveness of its production processes, and the comprehensive nature of its value evaluation system necessitate a tailored approach to understanding how policies influence enterprise behavior and performance (33).

Based on the above, several critical questions related to the TCM industry will be raised. Can TCMRIPs enhance enterprise performance and promote the development of the TCM industry? Should TCM enterprises increase their research and development investment in response to these policies to improve future performance? If performance can indeed be enhanced, what is the timeframe necessary to realize the maximum benefits, particularly considering the lag effects often associated with policy implementation and R&D outcomes? Current research does not provide definitive answers to these questions, highlighting a significant gap in our understanding of how to effectively utilize policy tools to stimulate the growth and innovation of the TCM industry. Consequently, scholars must undertake targeted analyses to provide evidence-based responses to these queries, which are essential for informing policy decisions, guiding enterprise strategies, and ultimately advancing the sustainable development of TCM.

## 2 Literature review and research hypotheses

### 2.1 The relationship between TCMRIPs and Enterprise performance

Innovation policies have long been a focal point of academic research, as they reshape enterprises’ competitive landscapes, drive industrial advancement, and have a profound impact on national economic growth (34). The existing literature provides robust evidence that innovation policies positively impact enterprise performance across diverse sectors and institutional settings (35). Within China’s transition toward an innovation-driven economy, the government has implemented a suite of innovation policies. Including R&D subsidies, tax incentives, intellectual property protection measures, and targeted regulatory reforms—aimed at enhancing enterprises’ innovation capabilities (36). These policies are widely acknowledged as critical drivers of increased innovation outputs, such as patent applications, new product launches, and technological breakthroughs, which ultimately translate into improved overall enterprise performance (37). R&D subsidy policies, as a form of government intervention, provide financial support to enterprises engaged in innovative activities. By mitigating the financial constraints associated with high-risk, long-term R&D projects—endeavors often plagued by information asymmetry and positive externalities that deter private investment—these subsidies enable enterprises to allocate more resources to research and development (38). This includes investments in advanced equipment, recruitment of specialized R&D personnel, and execution of extensive clinical trials or experimental studies. Consequently, enterprises can develop high-value-added new products, optimize production processes to reduce costs, and strengthen market competitiveness through technological differentiation, ultimately leading to improved financial performance, including higher revenues, enhanced profit margins, and increased market valuation (39). Tax incentives play a pivotal role in alleviating the tax burden on enterprises involved in innovative activities. These measures encompass super-deductions for R&D expenses, preferential tax rates for high-tech enterprises, and tax holidays for technology-driven startups. By effectively boosting post-tax profits, these incentives equip enterprises with additional resources that can be reinvested in R&D and other innovation-related initiatives (40). This influx of financial resources enables enterprises to attract top talent through competitive salaries and research grants, invest in cutting-edge technologies and state-of-the-art laboratory facilities, and conduct in-depth research on emerging technologies and market demands. Over the long term, these efforts contribute to enhanced innovation capabilities and improved enterprise performance. Within the context of the TCM industry, all TCM products in China must undergo a registration process before entering the market, with sales profits directly influencing an enterprise’s performance (41). This makes the investigation of the interplay between TCMRIPs and enterprise performance particularly crucial. Building on existing evidence and considering the unique context of TCM enterprises, we propose Hypothesis 1(a): TCMRIPs positively influence TCM enterprise performance. Specifically, TCMRIPs significantly enhance the performance of listed TCM enterprises in China. Notably, the impact of innovation policies on enterprise performance may not be immediate (36). Policy implementation typically involves a complex process, requiring enterprises to adjust their strategies, reorganize internal resources, and develop new capabilities to align with evolving policy frameworks (42). For example, enterprises may need to establish dedicated R&D teams to leverage policy incentives, collaborate with research institutions to meet new regulatory standards, or revise product development pipelines to align with policy objectives. Additionally, the outcomes of innovation activities stimulated by these policies, such as the development of new TCM products. This may take time to materialize, due to inherent uncertainties and lengthy processes in R&D, clinical trials, and market penetration (43). This delay stems from the time required for R&D investments to yield tangible results, for new products to complete regulatory approval, and for market acceptance to develop. In the TCM sector, this lag may be more pronounced due to the extensive documentation required for traditional usage, complex quality control standards for natural ingredients, and the gradual accumulation of clinical evidence supporting holistic therapeutic effects. Thus, we propose Hypothesis 1(b): The impact of TCMRIPs demonstrates a time lag, with their beneficial effects on enterprise performance becoming more pronounced over time and peaking after a specific duration.

*H1(a)*: TCMRIPs positively influence TCM enterprise performance.

*H1(b)*: The impact of TCMRIPs demonstrates a time lag

### 2.2 The relationship between TCMRIPs, R&D investment, and Enterprise performance

The interplay between policies, R&D investment, and enterprise performance has been extensively explored in academic research, with a growing body of evidence supporting the view that R&D investment acts as a critical mechanism through which innovation policies exert an impact on firm-level outcomes (44). Existing literature consistently demonstrates that well-designed policies can incentivize enterprises to augment their R&D expenditures, which in turn drives performance improvements, thereby forming a sequential chain of influence: policy → R&D investment → performance (45). Innovation policies, including R&D tax incentives and government-funded R&D subsidies, have been empirically validated as practical tools for boosting R&D investment across diverse industrial sectors. R&D tax incentives, such as tax credits, function by reducing the marginal cost of R&D activities for enterprises (46). When companies can benefit from lower tax rates on R&D expenditures or can deduct a specified percentage of their R&D costs from taxable income, the net cost of each additional R&D project diminishes. This creates a strong economic incentive for firms to allocate a greater share of resources to R&D initiatives, as the post-tax return on such investments becomes more appealing (47). On the other hand, government R&D subsidies provide direct financial support for research and development projects, spanning the entire innovation lifecycle from early-stage basic research to late-stage commercialization efforts. These subsidies play a pivotal role in alleviating financial constraints, particularly for small and medium-sized enterprises that often face challenges in accessing external capital markets (48). For TCM enterprises, investments in R&D domains, such as standardizing quality control protocols or conducting evidence-based clinical research, can also strengthen brand reputation and foster consumer trust, which in turn drives further performance improvements. Based on this conceptualization of the relationship between policy, R&D investment, and performance, we propose Hypothesis 2: TCMRIPs affect enterprise performance through the proportion of R&D investment, with R&D investment serving as a mediating variable in this relationship.

*H2*: TCMRIPs affect enterprise performance through the R&D investment.

### 2.3 Comparison between the newly version of the measures and previous versions

Policy effectiveness is not static; it evolves through revisions that reflect shifting industry demands, regulatory lessons, and societal expectations (49). In 2020, China issued the updated Drug Registration Administration Measures, representing a significant milestone in the nation’s drug regulatory framework. A comparison of the 2020 version with earlier iterations, such as the 2005 and 2007 versions, reveals that the new edition incorporates more targeted, refined, and industry-adapted policy measures, particularly regarding TCM (50). These enhancements are expected to enhance its effectiveness in improving enterprise performance. Firstly, the 2020 version integrates the achievements of reforms in the drug review and approval system over the past decade. It prioritizes innovative drugs and establishes a more science-driven evaluation system. The updated version clarifies the basic requirements for drug registration management, outlining the fundamental systems, principles, procedures, and key responsibilities of all involved parties in the process. This heightened transparency and predictability in the regulatory framework reduce uncertainties for enterprises, enabling them to plan their R&D and registration strategies more effectively and minimizing the risk of costly delays or rejections. Secondly, the 2020 version explicitly defines China’s TCM development path as focusing on “inheritance and innovation.” This dual emphasis addresses the unique needs of the TCM industry. By highlighting inheritance, the new version recognizes the value of traditional knowledge and classic formulas, simplifying registration requirements for products with well-documented traditional use. Simultaneously, it encourages innovation by promoting the application of modern scientific methods to validate the efficacy of TCM, develop new formulations, and expand clinical applications. This balanced approach provides enterprises with clear guidance on preserving traditional strengths while pursuing technological progress, thereby fostering a more supportive environment for R&D investment. Lastly, the classification of TCM registration has been significantly streamlined from nine categories to four, alleviating administrative burdens and optimizing the registration process. The previous classification system was criticized for its excessive complexity, featuring overlapping categories and ambiguous criteria that increased the time and costs associated with registration for TCM enterprises. The new classification—comprising innovative TCM, improved TCM, classic TCM prescriptions, and imported TCM—better aligns with the actual development needs of the industry, making it easier for enterprises to navigate the registration process and bring new products to market more rapidly. By reducing administrative barriers, aligning with industry characteristics, and providing clearer incentives for R&D and registration, the 2020 version is expected to create a more favorable policy environment that accelerates the translation of R&D efforts into market success. Therefore, we propose Hypothesis 3: The newly revised Drug Registration Administration Measures are more effective than previous versions in enhancing the performance of TCM listed enterprises.

*H3*: The newly version of the Drug Registration Administration Measures is more effective than previous versions in enhancing the performance of TCM listed enterprises.

## 3 Study design

### 3.1 Sample and data sources

TCM registration, review, and approval system operates under a supervisory framework composed of relevant laws and registration measures. The *Drug Registration Administration Measures*, first issued in 2002, have been updated three times, specifically in 2005, 2007, and 2020. Against this background, this study selects all A-share listed TCM enterprises in Shanghai and Shenzhen stock exchanges from 2002 to 2024 as the original sample. To ensure the accuracy and completeness of the conclusions, the original samples in this study were processed as follows: (1) Excluding ST/PT enterprises, as these entities typically adopt bankruptcy accounting standards, rendering them incomparable with other listed companies. Specific elimination steps: If the enterprise data in the dataset belongs to ST/PT in the current year, then in the downloaded data, the enterprise name will have the prefix “ST/PT” before it. Such data with the “ST/PT” prefix needs to be excluded. (2) Screening to include only enterprises specializing in proprietary Chinese medicines and TCM decoction pieces, while excluding those primarily engaged in TCM materials. This is because the TCM materials cannot be sold directly as products. They need to be further processed into Chinese herbal decoctions or Chinese patent medicines before they can be sold. (3) Excluding enterprises that received special treatment in the corresponding year (Contains missing values). (4) Perform winsorization at the 1% level to mitigate the impact of outliers. Enterprise-level data were sourced from the CSMAR database and the annual reports of individual companies (released at the end of each fiscal year). In this study, the relevant policies on the review and evaluation of all TCMRAPs issued by the Chinese government/NMPA are reviewed. The time period selected for this article is from 1 January 2002, to 31 December 2024. Policy texts were mainly retrieved and obtained from the official portal website of China’s central government,^1^ the Law and Regulation Database of Peking University,^2^ the China Food and Drug Administration),^3^ China National Knowledge Infrastructure,^4^ and other common retrieval platforms. In addition, relevant supplementary documents were searched from Baidu, Google, Bing, and other websites. The selected policy is related to the registration, review, and approval policy of TCM. In addition to “TCM registration,” “TCM review,” “TCM approval,” “New medicine review,” “Drug registration,” the search scope was also expanded by entering keywords such as “TCM innovation” to ensure a comprehensive and careful search. The types of policies retrieved included laws, ordinances, methods, rules and requirements, opinions, announcements, guiding principles, and notifications. Data analysis was conducted using Stata 18.0.

### 3.2 Definition of variables

#### 3.2.1 Dependent variable

Enterprise performance: Return on Assets (ROA) is selected to measure the performance of listed enterprises, calculated as net profit divided by the average balance of total assets (51). Note: In the robustness test, return on Equity (ROE) is used instead of ROA as the explained Variable (52).

#### 3.2.2 Independent variable

TCM Registration Policy Innovation Index (Pol): All Registration policies issued by the Chinese government from 2002 to 2024, covering four dimensions—TCM registration-related laws, registration measures & registration classifications, guiding principles, and notifications.

These policies were processed with stop-word removal and word segmentation. The top 300 words for each year were selected, and after manual screening, the frequency of innovation-oriented words was counted (53). The innovation-oriented keywords selected include, but are not limited to, the following words: innovate, new drug, clinical trial, entrance, digitize, data, develop, and others. After the screening process is completed, the team conducts a data review to prevent situations where inaccuracies in data screening occur due to individual differences in data interpretation. Finally, the annual frequency of innovation-oriented words in TCM registration policies multiplied by 1,000 is used to represent the TCM innovation policy.

#### 3.2.3 Mediating variable

R&D investment (RD): The logarithm of the R&D investment amount divided by 100. The logarithmic transformation was applied to R&D investment amount primarily to address its substantial range between maximum and minimum values, as well as its wide fluctuation. Logarithmic transformation effectively “compresses” the extreme values of the variable, mitigates the fluctuation amplitude of the high-value group, and thereby enhances the stability of the data variance. Regarding the division by 100, the purpose is to make the regression coefficients more suitable for comparison. These adjustments only alter the magnitude of the estimated coefficients, without changing the direction or significance level of the relationships between variables.

#### 3.2.4 Control variables

This study includes company size (Size), company age (Age), and asset-liability ratio (Lev) as control variables.

For all relevant variables and their definitions, please refer to Table 1.

**Table 1:** Relevant variables and definitions.

Variable type	Variable name	Variable symbol	Variable calculation rules
Dependent variable	Return on assets	ROA	(Net Income)/(Average Total Assets)
Dependent variable	Return on equity	ROE	(Net Income)/(Average Shareholders’ Equity)
Independent variable	TCM registration policy innovation index	Pol	Innovative word frequency * 1,000
Mediating variable	R&D investment	RD	Ln(Sum R&D investment)/100
Control variables	Enterprise size	Size	Ln(Total assets of the enterprise)
	Enterprise age	Age	Year of establishment of the enterprise
	Enterprise asset-liability ratio	Lev	Enterprise asset-liability ratio of the enterprise

### 3.3 Model construction

#### 3.3.1 Test model of the dependent variable and independent variable

In order to test the Dependent Variable and Independent Variable, multiple regression model (1) is constructed.


(1) $$\mathrm{RO}A_{i}=\alpha 0+\alpha 1\mathrm{Po}l_{i}+\text{contro}l_{i}+\sum \mathrm{Pro}+\varepsilon_{i}$$

In Equation 1, $\mathrm{RO}A_{i}$ is an explanatory variable indicating the enterprise performance, $\mathrm{Po}l_{i}$ is the TCM Registration Policy Innovation Index, and $\text{contro}l_{i}$ is a series of control variable coefficients. In addition, the fixed effects of enterprise is also controlled. $\varepsilon_{i}$ is the represents the disturbance term. α0 and α1 are the coefficient terms. If α1 significantly positive, it indicates that the TCMRIPs play a positive role in TCM listed enterprises performance.

Factors influencing firm performance typically also include R&D intensity, market competition level, macroeconomic fluctuations (e.g., GDP and pharmaceutical industry prosperity), and ownership structure, but these variables are not incorporated into the control variable set of this study for the following reasons: First, R&D intensity, as the mediating variable in this research, is specifically introduced only during the analysis of mediating effects rather than being included in control variables. Second, regarding market competition level, the traditional Chinese medicine industry features “high concentration in segmented markets,” where differences in market competition are mainly reflected in “variations in firm size”—the existing firm size variable has indirectly captured the level of market competition (i.e., larger firm scale corresponds to lower competitive intensity in the respective segmented market), and the two exhibit a significant substitution relationship, making redundant control unnecessary. Third, for macroeconomic fluctuations, this study has controlled for firm fixed effects, which can implicitly absorb common macroeconomic and industry-level shocks by offsetting performance fluctuations caused by “changes in macroeconomic conditions and industry cycles faced by the same firm across different years,” thus eliminating the need for additional macroeconomic variables. Finally, in terms of ownership structure, differences in policy treatments between state-owned and non-state-owned enterprises in the traditional Chinese medicine industry are partially reflected indirectly through firm size (since state-owned enterprises are mostly industry leaders with larger scales); moreover, the relatively balanced distribution of state-owned and non-state-owned enterprises in the study sample means that the impact of ownership structure on performance has been absorbed by firm fixed effects.

#### 3.3.2 Test model of the dependent variable, independent variable and mediating variable

In order to test the Dependent Variable, Independent Variable, multiple regression model (2) and model (3) are constructed.


(2) $$RD_{i}=\beta 0+\beta 1\mathrm{Po}l_{i}+\text{contro}l_{i,t}+\varepsilon_{i}$$

(3) $$\mathrm{RO}A_{i}=\gamma 0+\gamma 1\mathrm{Po}l_{i}+\gamma 2RD_{i}+\text{contro}l_{i}+\varepsilon_{i}$$

In Equations 2, 3, RD_i_ is a mediating variable indicating the R&D investment, $\mathrm{Po}l_{i}$ is the TCM Registration Policy Innovation Index, and $\text{contro}l_{i}$ is a series of control variable coefficients. $\varepsilon_{i}$ is the represents the disturbance term. β and γ are the coefficient terms. If β1 and γ1 both significantly positive, it indicates that R&D investment plays an intermediary role between TCMRIPs and TCM listed enterprises performance.

#### 3.3.3 Test model of the endogeneity test

In order to test the endogeneity issues model (4) is constructed.


(4) $$\mathrm{RO}A_{i}=\delta 0+\delta 1\mathrm{Po}l_{i}+\varepsilon_{i}$$

In Equation 4, $\mathrm{Po}l_{i}$ is the TCM Registration Policy Innovation Index, $\varepsilon_{i}$ is the represents the disturbance term. δ0 and δ1 are the coefficient terms. If δ1 significantly positive, it indicates that the selected TCMRIPs play a positive role in TCM listed enterprises performance.

## 4 Results

### 4.1 Descriptive statistics

Table 2 presents the descriptive statistics of the main variables in this study. The results indicate that the sample consists of 1,406 observations with no missing values for any variable. For the dependent Variable ROA, the Mean (0.067) and Median (0.066) are close, suggesting a relatively symmetric distribution of ROA. This indicates that the overall asset return level of enterprises is stable, with most firms exhibiting comprehensive asset utilization effects close to the average. Standard deviation (SD = 0.069) reflects a moderate degree of dispersion, indicating certain differences in asset returns among enterprises without extreme polarization. Extremes (Min = −0.549; Max = 0.355) means the negative minimum value indicates that some enterprises have negative asset returns (potentially facing operational difficulties or asset impairments), while the maximum value of 0.355 reflects high asset operational efficiency and prominent profitability in some enterprises. For Independent Variable Pol: The Median is close to the Mean, indicating an approximately symmetric distribution. Most observations show innovation-oriented word frequencies in TCM policies clustered around the Mean, with no significant skewness. A small standard deviation suggests low dispersion in Pol across the sample, reflecting either consistent emphasis on innovation in TCM registration policies from 2002 to 2024 (with minor annual/sample differences) or relatively balanced “exposure” to innovation-oriented policy terms among the sampled enterprises. For the First Control variable, Size, the Mean (21.356) and Median (21.404) are close, indicating a symmetric distribution of firm size. Most enterprises cluster around the 21.3–21.4 range, with no significant skewness in size differences within the industry. For the second Control variable, Age: Mean (17.312) and Median (17.000) are close, reflecting a symmetric distribution of firm age. The average establishment duration of sample enterprises is approximately 17 years, with most operating for around 17 years, indicating relative maturity in the industry. For the last Control Variables Lev: Mean (0.362) is slightly higher than the Median (0.329), suggesting a mild right-skewed distribution. This indicates the presence of some enterprises with high asset-liability ratios, which inflate the Mean; overall, the liability levels of sample enterprises remain within a reasonable range. All variables are deemed suitable for further empirical analysis.

**Table 2:** Descriptive statistics.

VarName	Obs	Mean	Median	SD	Min	Max
ROA	1,406	0.067	0.066	0.069	−0.549	0.355
pol	1,406	0.742	0.737	0.120	0.450	0.898
Lev	1,406	0.362	0.329	0.214	−0.224	1.579
Size	1,406	21.356	21.404	1.436	13.952	25.087
Age	1,406	17.312	17.000	7.280	0.000	43.000

### 4.2 Correlation analysis

As shown in Table 3, the correlation coefficient between ROA and Pol is significant at the 1% level with a positive sign, preliminarily validating Hypothesis 1(a). Specifically, a higher frequency of innovation-oriented terms in TCM policies (indicating stronger policy emphasis on innovation) is associated with a higher ROA. This may be explained by innovation-oriented policies that encourage the R&D of new TCM products and optimize registration processes, thereby enhancing enterprises’ product competitiveness and accelerating commercialization. Alternatively, policy resources may be allocated toward innovative enterprises, reducing operational costs and improving profitability. All control variables except Lev exhibit significant correlations with ROA at the 1% level. The non-significant correlation for Lev may stem from the complex relationship between capital structure and profitability in TCM enterprises. High-leverage firms face financial risks but may expand production, R&D, and other activities through debt financing, while low-leverage firms maintain stability but may be constrained by insufficient funds. These offsetting effects could weaken the linear relationship. Numerous domestic and foreign studies have confirmed that Lev has a certain impact on ROA. Therefore, in this study, this variable is still included in the control variables. Multiple covariance analysis (variance inflation factor, VIF) was conducted for all variables. As shown in Table 4, the VIF values of all independent variables are less than 10, indicating that there are no severe multiple covariance issues among the variables.

**Table 3:** Correlation analysis.

VarName	ROA	pol	Lev	Size	Age
ROA	1				
pol	0.076^***^	1			
Lev	−0.014	−0.151^***^	1		
Size	−0.177^***^	0.214^***^	−0.272^***^	1	
Age	−0.149^***^	0.435^***^	−0.176^***^	0.506^***^	1

*t* statistics in parentheses ^*^p < 0.1, ^**^p < 0.05, ^***^p < 0.01.

**Table 4:** Multiple covariance analysis.

VarName	VIF	1/VIF
Age	1.58	0.631946
Size	1.41	0.708731
pol	1.24	0.804650
Lev	1.09	0.917259
Mean VIF	1.33	

### 4.3 Basic regression analysis

#### 4.3.1 Hausman test

As shown in the Table 5, fixed-effect (Column 1), random-effect (Column 2), and OLS regressions (Column 3) were performed with ROA regressed on Pol and control variables. The resulting *p*-value is less than 0.000, indicating that the fixed-effect model should be selected for this set of variables. This confirms that the choice of Formula 1 is correct.

**Table 5:** Hausman test.

VarName	(1)	(2)	(3)
	FE	RE	OLS
pol	0.0960^***^	0.0922^***^	0.0984^***^
	(5.82)	(6.00)	(6.40)
Lev	−0.0180^**^	−0.000697	0.00467
	(−2.08)	(−0.07)	(0.50)
Size	−0.00709^***^	−0.0267^***^	−0.0164***
	(−4.83)	(−10.51)	(−8.62)
Age	−0.00148^***^	0.00155^***^	−0.000133
	(−4.83)	(3.30)	(−0.36)
_cons	0.179^***^	0.541^***^	0.344^***^
	(5.54)	(10.40)	(8.63)
*N*	1,406	1,406	1,406
*F*	24^***^	57^***^	
*r* ^2^ *P*	0.063	0.147 0.000	

*t* statistics in parentheses ^*^p < 0.1, ^**^p < 0.05, ^***^p < 0.01.

#### 4.3.2 The relationship between policies and Enterprise performance

To verify the effect of Pol on ROA, regression analysis was conducted on Model 1, and the results are presented in Table 6. Column (1) includes all control variables, while Column (2) does not include any control variables. It can be seen that, regardless of whether control variables are included, the impact of Pol on ROA is positive and significant at the 1% level. The correlation coefficients between A and B are 0.0873 and 0.0350. This suggests that the frequency of innovation-oriented terms in policies positively impacts enterprise performance, thereby validating Hypothesis 1(a).

**Table 6:** Benchmark regression analysis.

VarName	(1)	(2)
	ROA	ROA
pol	0.0873^***^	0.0350^***^
	(6.47)	(2.83)
Lev	0.00717	
	(0.77)	
Size	−0.0270^***^	
	(−11.30)	
Age	0.00178^***^	
	(4.17)	
_cons	0.546^***^	0.0412^***^
	(11.21)	(4.43)
Stkcd	Yes	Yes
*N*	1,406	1,406
*F*	65^***^	8^***^
*r* ^2^	0.402	0.288

*t* statistics in parentheses ^*^p < 0.1, ^**^p < 0.05, ^***^p < 0.01.

#### 4.3.3 The relationship between policies, R&D investment, and Enterprise performance

Column (1) in the Table 7 shows the results of the normal fixed-effect model calculated by Formula 1, and Column (2) presents the mediation effect model calculated by Model (2, 3) (with the mediating variable RD as the explained variable, Pol as the explanatory variable, and control variables included in the regression). It is observed that the coefficient of Pol on the mediating variable RD is positive and significant at the 1% level. Then, adding the mediating variable RD to the main regression model is shown in Column (3), where all coefficients are significant and positive, indicating that the mediating variable RD plays a partial mediating role between Pol and ROA, thus validating Hypothesis 2.

**Table 7:** Mediated effects test.

VarName	(1)	(2)	(3)
	ROA	RD	ROA
pol	0.0873^***^	0.0718^***^	0.0615^***^
	(6.47)	(13.40)	(3.55)
RD			0.480^***^
			(5.92)
Lev	0.00717	0.00447	−0.0202^**^
	(0.77)	(1.59)	(−2.35)
Size	−0.0270^***^	0.00612^***^	−0.0100^***^
	(−11.30)	(12.81)	(−6.54)
Age	0.00178^***^	0.000461^***^	−0.00171^***^
	(4.17)	(4.62)	(−5.58)
_cons	0.546^***^	−0.0254^**^	0.191^***^
	(11.21)	(−2.42)	(5.98)
*N*	1,406	1,406	1,406
*F*	65^***^	182^***^	26^***^
*r* ^2^	0.402	0.342	0.086

*t* statistics in parentheses ^*^p < 0.1, ^**^p < 0.05, ^***^p < 0.01.

#### 4.3.4 Heterogeneity test by year grouping

Regression analysis was conducted on enterprise ROA, Pol, and control variables for the period 2020–2024; however, the results were insignificant, rendering a comparative analysis impossible. The reason may be attributed to the short time period (2020–2024) and a small sample size (*N* = 378), which hindered effective data fitting and the accurate reflection of real-world relationships. Another more important reason is that during this period, COVID-19 broke out globally, leading to abnormal data for enterprises. Therefore, to make a more accurate comparison, we compared the overall data from 2002 to 2024 with that from 2002 to 2019, and indirectly analyzed the pros and cons of the new policy. As shown in Table 8, Column (1), a comparative analysis was performed between the regression results of enterprise ROA, Pol, and control variables for 2002–2024 (the main regression of this study). Moreover, as shown in Table 8, Column (2), a comparative analysis was performed between the regression results of enterprise ROA, Pol, and control variables for 2002–2019. Both results were significant at the 1% level, further indicating that innovation in policies during both periods significantly improved enterprise performance. A further comparative analysis revealed that for 2002–2019, the coefficient of Pol on enterprise performance was 0.688, which is statistically significant at the 1% level. This indicates a highly significant positive relationship between ROA and Pol. While for 2002–2024, the coefficient was 0.0922. This suggests that including data from 2020 to 2024 strengthens the impact of policy-related term frequency on ROA, implying that policies implemented after the 2020 reform are more effective in improving enterprise performance and that the new version of the policy is superior to the old one, validating Hypothesis 3.

**Table 8:** Heterogeneity analysis.

VarName	(1)	(2)
	ROA (2002–2024)	ROA (2002–2019)
pol	0.0873^***^	0.0688^***^
	(6.47)	(3.88)
Lev	0.00717	0.00665
	(0.77)	(0.66)
Size	−0.0270^***^	−0.0284^***^
	(−11.30)	(−10.59)
Age	0.00178^***^	0.00335^***^
	(4.17)	(5.29)
_cons	0.546^***^	0.571^***^
	(11.21)	(10.31)
Stkcd	Yes	Yes
*N*	1,406	1,028
*F*	65^***^	52^***^
*r* ^2^	0.402	0.487

*t* statistics in parentheses ^*^p < 0.1, ^**^p < 0.05, ^***^p < 0.01.

Enterprises are categorized into small and large groups based on the median value: those with values below the median are defined as small enterprises, while those at or above the median are classified as large enterprises.

As presented in Column (1) of Table 9, a comparative analysis was conducted on the regression results of ROA, Pol, and control variables for small enterprises spanning the period 2002–2024. Additionally, Column (2) of Table 9 reports a comparative analysis of the same set of variables (ROA, Pol, and control variables) for small enterprises during 2002–2019. Both sets of results are statistically significant at the 1% level, further confirming that policy innovations in both periods significantly enhanced the performance of small enterprises. A more detailed comparison reveals that for the 2002–2019 period, the coefficient of Pol on enterprise performance is 0.0934, whereas this coefficient rises to 0.117 for 2002–2024. This indicates that incorporating data from 2020 to 2024 amplifies the impact of policy-related term frequency on ROA, suggesting that policies implemented post the 2020 reform are more effective in boosting the performance of small enterprises.

**Table 9:** Grouped regression analysis.

VarName	(1)	(2)	(3)	(4)
	ROA (2002–2024 Small size)	ROA (2002–2019 Small size)	ROA (2002–2024 Big size)	ROA (2002–2019 Big size)
pol	0.117^***^	0.0934^***^	0.0556^***^	0.0524^***^
	(5.35)	(3.68)	(3.29)	(2.16)
Lev	−0.0134	−0.0186	−0.0381^***^	−0.00980
	(−0.97)	(−1.25)	(−2.03)	(−0.45)
Size	−0.0339^***^	−0.0345^***^	−0.0178^***^	−0.0353^***^
	(−9.41)	(−8.77)	(−3.09)	(−5.02)
Age	−0.000504	0.00121	−0.000316	0.00294^**^
	(−0.68)	(1.24)	(−0.44)	(2.52)
_cons	0.685^***^	0.696^***^	0.442^***^	0.775^***^
	(9.28)	(8.60)	(3.80)	(5.40)
Stkcd	Yes	Yes	Yes	Yes
*N*	702	601	691	423
*F*	53^***^	39^***^	13^***^	11^***^
*r* ^2^	0.516	0.556	0.492	0.619

*t* statistics in parentheses ^*^p < 0.1, ^**^p < 0.05, ^***^p < 0.01.

For large enterprises, Column (3) of Table 9 reports the comparative analysis of regression results for their ROA, Pol, and control variables over 2002–2024, while Column (4) focuses on the 2002–2019 period. Both sets of results were also significant at the 1% level, indicating that policy innovations in these two periods similarly improved the performance of large enterprises. A further breakdown shows that the coefficient of Pol on large enterprise performance was 0.0524 (statistically significant at the 1% level) during 2002–2019, and rose slightly to 0.0556 for 2002–2024.

A cross-group comparison of the increase in the Pol coefficient between small and large enterprises reveals that, in terms of performance improvement, the post-2020 reform policies exert a more pronounced effect on small enterprises.

## 5 Endogeneity test and robustness test

### 5.1 Endogeneity test

To avoid potential bidirectional causality between ROA and Pol, this study conducts an endogeneity test using lagged periods of the explained variable with model (4). Specifically, while Pol in a given year may affect the current year’s ROA (as indicated by the strong positive correlation in the regression equation), it is also possible that the current year’s ROA influences the current year’s Pol. Therefore, a forward-looking regression analysis was conducted on the ROA, given that ROA in a given year cannot affect Pol in the previous year. As shown in Table 10, a 0–3 period forward-looking regression analysis was conducted on the ROA. The results indicate that, except for the significant correlation between the current year’s ROA and Pol in Column (1), ROA fronted by 1–3 periods in Column (2–4) shows no correlation with Pol. This confirms that there is no strong endogeneity among the variables selected in this study. However, Companies with higher levels of profitability can invest more in R&D, regardless of policy. Therefore, in this paper, a two-stage system GMM is also used to recheck and verify the correlation between the dependent variable and the independent variables. The GMM uses lagged 1-period and 2-period ROA as proxies to construct the dynamic panel, and uses the lagged period polynomial as an instrument variable. As shown in Table 11, AR(1) (0.000) and AR(2) (0.127) indicate that the first-order difference exhibits autocorrelation, while the second-order difference does not. Therefore, the GMM estimation is a practical approach. The Hansen J test statistic (0.238) is not significant, indicating that the model does not suffer from over-identification, the instrumental variables are valid, and the model set-up is reasonable. The results show that, after considering the possible endogenous problems, the promotion effect of TCMRIPs on the performance increase of TCM listed enterprises remains established, and the regression results are positively correlated at the 5% significance level.

**Table 10:** Lagged periods Endogeneity test.

VarName	(1)	(2)	(3)	(4)
	ROA	L. ROA	L2. ROA	L3. ROA
pol	0.0435^***^	0.0155	0.0207	−0.00592
	(2.86)	(1.02)	(1.28)	(−0.31)
_cons	0.0343^***^	0.0584^***^	0.0565^***^	0.0777^***^
	(3.00)	(5.06)	(4.58)	(5.28)
*N*	1,406	1,318	1,236	1,156
*F*	8^***^	1	2	0
*r* ^2^	0.006	0.001	0.001	0.000

*t* statistics in parentheses ^*^p < 0.1, ^**^p < 0.05, ^***^p < 0.01.

**Table 11:** Two-stage system GMM regression test.

VarName	ROA
L. ROA	0.590^***^
	(7.45)
L2. ROA	0.0650
	(0.75)
pol	0.0395^**^
	(2.02)
RD	0.0585
	(0.29)
Size	0.000350
	(0.12)
Age	−0.00711^**^
	(−1.96)
Lev	−0.0577^***^
	(3.48)
_cons	−0.0358
	(−0.94)
*N*	1,232
AR(1)	0.000
AR(2)	0.127
Hansen test	0.238

*t* statistics in parentheses ^*^p < 0.1, ^**^p < 0.05, ^***^p < 0.01.

### 5.2 Robustness test

A fixed-effect model regression was performed using ROE (Return on Equity) instead of ROA, with Pol and control variables included. The results remained significant, confirming robustness. As shown in Table 12, the empirical analyses of the previous study are robust.

**Table 12:** Robustness test.

ROE	Coefficient	Std. err.	*t*	*P* > |*t*|	[95% conf. interval]	
pol	0.1420154	0.0237323	5.98	0.000	0.0954583	0.1885724
Lev	0.0714547	0.0162781	4.39	0.000	0.0395209	0.1033884
Size	−0.0484834	0.0042054	−11.53	0.000	−0.0567335	−0.0402334
Age	0.0036284	0.000752	4.83	0.000	0.0021533	0.0051036
_cons	0.939657	0.0856298	10.97	0.000	0.7716721	1.107642
*F*	86.24					
*N*	1,406					
Adj *R*^2^	0.3199					
*P*	0.0000					
Freedom (Stkcd)	78					

## 6 Heterogeneity analysis

Studies have shown that policies may continue to affect various enterprise indicators for a period after their issuance. Therefore, Pol was fronted by three periods using model (1), and as shown in Table 13, the coefficient reached its maximum at the third front. This suggests that the effect of policy issuance peaks after 3 years: enterprises increase performance by raising R&D investment, and this positive relationship leads to maximum enterprise performance 3 years later, validating Hypothesis 1(b).

**Table 13:** Regression with fronted periods of Pol.

	(1)	(2)	(3)	(4)
	ROA	ROA	ROA	ROA
pol	0.0922^***^			
	(6.00)			
L1_pol		0.104^***^		
		(5.02)		
L2_pol			0.113^***^	
			(5.52)	
L3_pol				0.0842^***^
				(4.23)
Lev	−0.000697	0.00224	−0.00461	−0.0393^**^
	(−0.07)	(0.19)	(−0.33)	(−2.52)
Size	−0.0267^***^	−0.0295^***^	−0.0272^***^	−0.0130^***^
	(−10.51)	(−10.03)	(−7.77)	(−3.11)
Age	0.00155^***^	0.00125^**^	0.000794	−0.000547
	(3.30)	(2.07)	(1.24)	(−0.82)
_cons	0.541^***^	0.598^***^	0.555^***^	0.302^***^
	(10.40)	(9.89)	(7.92)sss	(3.74)
*N*	1,406	1,318	1,236	1,156
*F*	57^***^	47^***^	29^***^	11^***^
*r* ^2^	0.367	0.361	0.343	0.345

*t* statistics in parentheses ^*^p < 0.1, ^**^p < 0.05, ^***^p < 0.01.

## 7 Conclusion and recommendations of the study

### 7.1 Conclusion

This study conducts an empirical analysis on the data of A-share TCM enterprises listed on the Shanghai and Shenzhen Stock Exchanges from 2002 to 2024 by constructing an unbalanced panel fixed-effect model, aiming to explore the impact of TCMRIPs on enterprise performance. The results show that there is a significant positive correlation between TCMRIPs and the performance of TCM listed enterprises in China, which strongly proves that innovation-oriented policies have a positive regulatory effect on enterprise performance. This means that TCMRIPs can effectively promote enterprises to improve their performance through a series of mechanisms, such as encouraging the research and development of new drugs and optimizing the registration process. The study finds that the impact of the policy on enterprise performance has a lag, and it takes a certain period of time for the policy effect to be fully manifested. According to the results, the positive impact of the policy on the enterprise’s performance reaches its peak in the third year. From the perspective of the relationship among policies, R&D investment, and enterprise performance, the study confirms that TCMRIPs affect enterprise performance by influencing the proportion of enterprises’ R&D investment. That is, policies can encourage enterprises to increase R&D investment, and the increase in R&D investment is positively correlated with the improvement of enterprise performance. This indicates that R&D investment plays a key mediating role between policies and enterprise performance.

In addition, beyond the statistical finding that TCMRIPs exert a significant positive lagged effect—with this impact peaking in the third year—and that R&D investment serves as a critical mediating factor, TCMRIPs ultimately influence enterprise performance through three closely intertwined mechanisms. First, as a formal institutional arrangement under the umbrella of industrial policy, TCMRIPs effectively mitigate market uncertainty and information asymmetry. For instance, the new version of the Drug Registration Administration Measures clarifies the development path for TCM and simplifies registration classification, which sends unambiguous policy signals to enterprises. These signals help enterprises avoid blind investment in R&D projects that do not align with policy priorities, while also enhancing their access to more stable external financing (e.g., equity investment or credit support), as investors gain greater confidence in the industry’s policy-backed development prospects. Second, TCMRIPs drive adjustments to enterprises’ internal resource allocation. Backed by implicit incentives such as accelerated registration for innovative TCM drugs and potential policy preferences (e.g., tax breaks or subsidies), enterprises reallocate resources away from low-value-added production of mature products toward high-value R&D activities. This realignment of resources directly aligns with a core goal of industrial policy: promoting industrial upgrading by optimizing resource distribution in strategically important sectors. Third, TCMRIPs facilitate the conversion of R&D achievements into tangible enterprise performance. Shortened registration cycles enable innovative TCM drugs to enter the market more quickly, allowing enterprises to recoup R&D costs promptly and secure first-mover advantages in competitive segments. This mechanism-centered analysis not only clarifies why the statistical correlation between TCMRIPs and enterprise performance exists but also embeds the study’s empirical findings within the broader theoretical framework of industrial policy. In doing so, it strengthens the research’s theoretical depth and enhances its explanatory power for both academic and practical audiences.

### 7.2 Discussion

Although this study verifies the positive impact of the TCMRIPs on corporate performance and the mediating role of R&D investment through basic regression, endogeneity tests, and robustness tests using ROE instead of ROA, with clear logic in statistics and correlation analysis, there remains room for deepening the interpretation of the correlation between control variables (Size, Age, and Lev) and corporate performance as well as policy effects. The existing research only incorporates these three variables into the model as independent control variables to eliminate interference, without fully analyzing their own mechanism of action on corporate performance and the potential correlation with policy effects. Therefore, we need to separately interpret and discuss the results of these three variables.

For Size, as a core reflection of the resource endowment of TCM enterprises, firm size does not have a simple linear correlation with corporate performance. Instead, it exerts an indirect influence through the chain of “resource integration capability—policy response efficiency—performance conversion” and has implicit adaptability to the effects of TCMRIPs.

From the perspective of the basic support for corporate performance, the sample data shows that the mean value of Size is 21.356 and the median is 21.404, with a symmetric and concentrated distribution. This indicates that most listed TCM enterprises are at a medium scale in the industry, and there is no extreme differentiation in size. This scale characteristic determines the enterprises’ ability to acquire and utilize resources: large enterprises usually have more complete R&D infrastructure, wider market channels, and stronger compliance management capabilities. Under the guidance of TCMRIPs, large enterprises can quickly convert policy support into practical actions. TCM R&D is characterized by high investment, long cycles, and high uncertainty. Although TCMRIPs can reduce enterprises’ R&D costs through subsidies, tax reductions, and other means, they cannot completely eliminate risks such as technical failures and low market acceptance. With sufficient cash flow reserves, large enterprises can withstand the losses from a single R&D failure and continue to invest in innovative projects in line with policy orientation. In contrast, if small enterprises experience R&D failures, they may face the risk of capital chain rupture and be forced to terminate R&D activities related to policy response, thereby weakening the role of policies in promoting performance.

For Age, which reflects the industry experience and operational maturity of TCM enterprises. Its impact on corporate performance has a “double-edged sword” characteristic and also indirectly affects enterprises’ response efficiency to TCMRIPs. The sample data shows that the mean value of Age is 17.312 and the median is 17.000, indicating that most sample enterprises have been established for more than 15 years and are in the mature stage of operation. Long-term industry involvement has enabled these enterprises to accumulate advantages in three aspects: first, experience in traditional technologies and formulas. The core competitiveness of TCM enterprises is often related to classic prescriptions and traditional processing techniques. Older enterprises have deeper technical accumulation in these fields and can develop improved new drugs (such as the secondary development of classic famous prescriptions) based on traditional advantages, reducing R&D risks. Second, accumulation of market trust. Through long-term product quality control, older enterprises have formed high brand awareness among consumers. After newly registered products are launched, they are more likely to gain market acceptance, accelerating performance conversion. Third, experience in policy adaptation. From the first version of the Measures for the Administration of Drug Registration in 2002 to the new version in 2020, older enterprises have gone through multiple rounds of policy adjustments, making them more sensitive to changes in registration processes and approval standards. They can quickly adjust R&D and declaration strategies to avoid performance losses caused by insufficient policy adaptation.

For Lev, the result that the asset-liability ratio has no significant correlation with corporate performance is the most important part requiring in-depth interpretation in the analysis of control variables. The sample data shows that the mean value of Lev is 0.362 and the median is 0.329, presenting a slightly right-skewed distribution. This indicates that there are some enterprises with high liabilities, but the overall liability level is within a reasonable range (the reasonable range of asset-liability ratio in the pharmaceutical industry is generally considered to be 30–50%). This result actually stems from the “offset of two-way effects” and “scenario dependence” of liabilities on performance, and there is an implicit interaction with the role of TCMRIPs.

From the perspective of the “offset of two-way effects,” the impact of liabilities on the performance of TCM enterprises has both positive and negative effects: on the one hand, moderate liabilities can supplement R&D and operating funds to help enterprises respond to TCMRIPs. For example, enterprises can expand R&D teams and conduct clinical trials through bank loans, or invest funds to optimize registration materials to meet the requirements of the new policies, thereby promoting performance improvement. On the other hand, excessive liabilities will increase financial risks and squeeze the space for R&D investment. TCM R&D has a long cycle. If an enterprise has a too high liability level, it needs to give priority to repaying interest, which may lead to the misappropriation of R&D funds, or even the termination of innovative projects in line with policy orientation due to short-term debt repayment pressure, dragging down performance. The slightly right-skewed distribution of Lev in the sample means that the negative effects of some high-liability enterprises offset the positive effects of most enterprises with reasonable liabilities, ultimately resulting in no significant correlation between Lev and ROA in the linear model.

From the perspective of “scenario dependence,” the impact of Lev on performance is also related to the “purpose of liabilities” and “policy adaptability” of enterprises. If enterprises use debt funds for R&D activities encouraged by TCMRIPs, liabilities may be converted into performance through policy dividends. If liabilities are only used for the expansion of traditional production, it will be difficult to enjoy policy support, and performance may decline due to increased market competition. The existing model does not distinguish between “liability purposes,” making it impossible to capture this scenario difference. For example, after the implementation of the 2020 new version of the *Measures for the Administration of Drug Registration*, some enterprises raised funds to carry out R&D of “innovative TCM drugs,” and such liabilities were positively correlated with performance. In contrast, some enterprises still raised funds to expand the production capacity of TCM decoction pieces, and such liabilities were negatively correlated with performance. The combination of these two situations leads to the non-significance of the Lev coefficient. In addition, the impact of Lev may also vary by enterprise size: large enterprises are more likely to obtain R&D funds through liabilities and have strong debt repayment capabilities, so liabilities may be positively correlated with performance; small enterprises mostly use liabilities for operating capital turnover, which may be negatively correlated with performance. This size difference further exacerbates the “apparent no correlation” between Lev and performance.

### 7.3 Recommendations

Based on the above conclusions, we can put forward the following recommendations:

(1) For the government

Continuous efforts should be directed toward promoting innovation in the registration of TCM while further streamlining procedural simplifications. Considering that the newly version of the Drug Registration Administration Measures has proven to be highly effective in enhancing enterprise performance, it is essential for the government to ensure continuity and stability in these policy refinements. This approach can significantly boost enterprise performance, which can then translate into improved products through various avenues such as upgrading product quality, expanding product lines, and enhancing clinical efficacy, thus fostering the advancement of TCM. Moreover, given the lag effect of policies, with their positive impacts peaking in the third year, the government should establish a robust long-term policy implementation and evaluation mechanism. It is essential to avoid short-term policy fluctuations, ensuring that enterprises have stable policy expectations to facilitate long-term R&D planning. Concurrently, policies should be dynamically adjusted based on the actual development of the TCM industry. This could involve regularly collecting feedback from enterprises regarding the effects of policy implementation, tracking changes in R&D investments, and monitoring the performance of listed TCM enterprises. Such information can help modify and enhance relevant policies in a timely manner to better meet the needs of industrial development. Additionally, since TCMRIPs influence enterprise performance through R&D investment, the government could introduce supportive measures to encourage enterprises to increase their R&D expenditures. For example, this could include providing tax incentives for R&D costs. Such as increasing the percentage of R&D expense deductions. Or establishing special funds to subsidize R&D projects for TCM enterprises, particularly small and medium-sized enterprises with relatively limited R&D capabilities. These initiatives could further amplify the role of R&D investment as a mediating factor, thereby enhancing the positive impact of policies on enterprise performance.

(2) For enterprises:

Policies have the potential to significantly enhance enterprise performance, with their effects often reaching a peak after 3 years. Consequently, businesses should develop long-term R&D investment strategies that align with the policy cycle. Specifically, enterprises should consider increasing their R&D investment intensity in line with their own development cycles. This approach can improve enterprise performance, boost output, and further advance the growth of the TCM industry. Enterprises must recognize the crucial mediating role that R&D investment plays and incorporate it into their core development strategies. Establishing a dedicated R&D management department can help conduct comprehensive research on trends in TCM new drug development and policy guidance. It is essential to formulate scientific R&D plans to ensure the effective allocation of R&D funds. Additionally, given the inherent lag in policy effects, enterprises should exhibit patience and persistence in their R&D investments. They should avoid reducing or halting R&D funding due to short-term performance fluctuations and instead commit to long-term investments, allowing sufficient time for the maximum impact of policies to materialize.

## Data Availability

The original contributions presented in the study are included in the article/Supplementary material, further inquiries can be directed to the corresponding author.

## Footnotes

1
https://www.gov.cn/

2
https://www.pkulaw.com/

3
https://www.nmpa.gov.cn/

4
https://www.cnki.net/

## References

[1] Cyranoski D. Why Chinese medicine is heading for clinics around the world. Nature. (2018) 561:448–50. doi: 10.1038/d41586-018-06782-7, PMID: 30258149

[2] Chan K Hu X-Y Razmovski-Naumovski V Robinson N. Challenges and opportunities of integrating traditional Chinese medicine into mainstream medicine: a review of the current situation. Eur J Integr Med. (2015) 7:67–75. doi: 10.1016/j.eujim.2014.12.006

[3] Griffiths S. Integrating traditional Chinese medicine: experiences from China. Australas Med J. (2010) 3:385–96. doi: 10.4066/AMJ.2010.411

[4] Wu W-Y Hou J-J Long H-L Yang W-Z Liang J Guo D-A. TCM-based new drug discovery and development in China. Chin J Nat Med. (2014) 12:241–50. doi: 10.1016/S1875-5364(14)60050-9, PMID: 24863348

[5] Xu J Xia Z. Traditional Chinese medicine (TCM) – does its contemporary business booming and globalization really reconfirm its medical efficacy & safety? Med Drug Discov. (2019) 1:100003. doi: 10.1016/j.medidd.2019.100003

[6] Alostad AH Steinke DT Schafheutle EI. A qualitative exploration of Bahrain and Kuwait herbal medicine registration systems: policy implementation and readiness to change. J Pharm Policy Pract. (2019) 12:32. doi: 10.1186/s40545-019-0189-7, PMID: 31624636 PMC6784343

[7] Kuang Z Li X Cai J Chen Y Qiu X Ni X . Calling for improved quality in the registration of traditional Chinese medicine during the public health emergency: a survey of trial registries for COVID-19, H1N1, and SARS. Trials. (2021) 22:188. doi: 10.1186/s13063-021-05113-y, PMID: 33673845 PMC7934977

[8] Dai W Zhong M Lin W Su L. Overview on the amendments of provisions for drug registration in China. J Clin Pharmacol. (2021) 61:74–81. doi: 10.1002/jcph.1700, PMID: 32656769

[9] Parry J. Queue for drug registrations grows longer in China. BMJ. (2015) 350 13:h1596–6. doi: 10.1136/bmj.h1596, PMID: 25800969

[10] Green A Lyus R Ocan M Pollock A Brhlikova P. Registration of essential medicines in Kenya, Tanzania and Uganda: a retrospective analysis. J R Soc Med. (2023) 116:331–42. doi: 10.1177/01410768231181263, PMID: 37343667 PMC10695152

[11] Phillips AT Desai NR Krumholz HM Zou CX Miller JE Ross JS. Association of the FDA amendment act with trial registration, publication, and outcome reporting. Trials. (2017) 18:333. doi: 10.1186/s13063-017-2068-3, PMID: 28720112 PMC5516301

[12] Naudet F Siebert M Boussageon R Cristea IA Turner EH. An open science pathway for drug marketing authorization—registered drug approval. PLoS Med. (2021) 18:e1003726. doi: 10.1371/journal.pmed.1003726, PMID: 34370737 PMC8351924

[13] Qu L Zou W Wang Y Wang M. European regulation model for herbal medicine: the assessment of the EU monograph and the safety and efficacy evaluation in marketing authorization or registration in member states. Phytomedicine. (2018) 42:219–25. doi: 10.1016/j.phymed.2018.03.048, PMID: 29655689

[14] Lu D Dou F Qi F Gao J. Tailored to fit: China optimizes policies and regulations regarding drug registration and review to promote innovation in traditional Chinese medicine. Drug Discov Ther. (2024) 18:210–2. doi: 10.5582/ddt.2024.01055, PMID: 39034115

[15] Fan X Meng F Wang D Guo Q Ji Z Yang L . Perceptions of traditional Chinese medicine for chronic disease care and prevention: a cross-sectional study of Chinese hospital-based health care professionals. BMC Complement Altern Med. (2018) 18:209. doi: 10.1186/s12906-018-2273-y, PMID: 29976190 PMC6034332

[16] Liang B Gu N. Traditional Chinese medicine for coronary artery disease treatment: clinical evidence from randomized controlled trials. Front Cardiovasc Med. (2021) 8:702110. doi: 10.3389/fcvm.2021.702110, PMID: 34422929 PMC8377193

[17] Li L Yao H Wang J Li Y Wang Q. The role of Chinese medicine in health maintenance and disease prevention: application of constitution theory. Am J Chin Med. (2019) 47:495–506. doi: 10.1142/S0192415X19500253, PMID: 31023059

[18] Liang Z Lai Y Li M Shi J Lei CI Hu H . Applying regulatory science in traditional chinese medicines for improving public safety and facilitating innovation in China: a scoping review and regulatory implications. Chin Med. (2021) 16:23. doi: 10.1186/s13020-021-00433-2, PMID: 33593397 PMC7884970

[19] Li Q Wang H Li X Zheng Y Wei Y Zhang P . The role played by traditional Chinese medicine in preventing and treating COVID-19 in China. Front Med. (2020) 14:681–8. doi: 10.1007/s11684-020-0801-x, PMID: 32651936 PMC7348116

[20] Liang B Zhu Y-C Lu J Gu N. Effects of traditional Chinese medication-based bioactive compounds on cellular and molecular mechanisms of oxidative stress. Oxidative Med Cell Longev. (2021) 2021:3617498. doi: 10.1155/2021/3617498, PMID: 34093958 PMC8139859

[21] Bouet D. A study of intellectual property protection policies and innovation in the Indian pharmaceutical industry and beyond. Technovation. (2015) 38:31–41. doi: 10.1016/j.technovation.2014.10.007

[22] Liu S Zhu J Li J. The interpretation of human body in traditional Chinese medicine and its influence on the characteristics of TCM theory. Anat Rec. (2021) 304:2559–65. doi: 10.1002/ar.24643, PMID: 34117702

[23] Liu Q Huang Z Mao Z. Has the consistency evaluation policy of generic drugs promoted the innovation quality of Chinese pharmaceutical manufacturing industry? An empirical study based on the difference-in-differences model. Front Public Health. (2023) 11:1265756. doi: 10.3389/fpubh.2023.1265756, PMID: 38106910 PMC10722264

[24] Liu H Ma Z. Analysis on situation of traditional Chinese medicine development and protection strategies in China. Chin J Integr Med. (2020) 26:943–6. doi: 10.1007/s11655-020-3218-0, PMID: 32623703

[25] Zhao Z Guo P Brand E. The formation of daodi medicinal materials. J Ethnopharmacol. (2012) 140:476–81. doi: 10.1016/j.jep.2012.01.048, PMID: 22342382

[26] Liu H-B Li B Guo LW Pan LM Zhu HX Tang ZS . Current and future use of membrane Technology in the Traditional Chinese Medicine Industry. Sep Purif Rev. (2022) 51:484–502. doi: 10.1080/15422119.2021.1995875

[27] Chan K. Chinese medicinal materials and their interface with Western medical concepts. J Ethnopharmacol. (2005) 96:1–18. doi: 10.1016/j.jep.2004.09.019, PMID: 15588645

[28] Song S Qiu R Jin X Zhou Z Yan J Ou Q . Mechanism exploration of ancient pharmaceutic processing (Paozhi) improving the gastroprotective efficacy of Aucklandiae Radix. J Ethnopharmacol. (2022) 287:114911. doi: 10.1016/j.jep.2021.114911, PMID: 34902533

[29] Lu L Zeng J. Inequalities in the geographic distribution of hospital beds and doctors in traditional Chinese medicine from 2004 to 2014. Int J Equity Health. (2018) 17:165. doi: 10.1186/s12939-018-0882-1, PMID: 30419919 PMC6233493

[30] Singh K Gupta JK Jain D Kumar S Singh T Saha S. Exploring the ancient wisdom and modern relevance of Chinese medicine: a comprehensive review. Pharmacol Res Mod Chin Med. (2024) 11:100448. doi: 10.1016/j.prmcm.2024.100448

[31] Dubey RK Kumar B. A comprehensive overview of traditional Chinese medicine (TCM). Chin J Appl Physiol. (2025) 41:e20250015. doi: 10.62958/j.cjap.2025.015, PMID: 40605540

[32] Li Z-G Wei H. A comprehensive evaluation of China’s TCM medical service system: an empirical research by integrated factor analysis and TOPSIS. Front Public Health. (2020) 8:532420. doi: 10.3389/fpubh.2020.532420, PMID: 33117767 PMC7550738

[33] Moorhouse TP Zhou Z Ye Y Zhou Y D’Cruze NC Macdonald DW. What is ‘TCM’? A conservation-relevant taxonomy of traditional Chinese medicine. Glob Ecol Conserv. (2021) 32:e01905. doi: 10.1016/j.gecco.2021.e01905, PMID: 41237400

[34] Mazzucato M. Mission-oriented innovation policies: challenges and opportunities. Ind Corp Change. (2018) 27:803–15. doi: 10.1093/icc/dty034

[35] Xie X Zeng S Peng Y Tam C. What affects the innovation performance of small and medium-sized enterprises in China? Innovation. (2013) 15:271–86. doi: 10.5172/impp.2013.15.3.271

[36] Wang S Ahmad F Li Y Abid N Chandio AA Rehman A. The impact of industrial subsidies and Enterprise innovation on Enterprise performance: evidence from listed Chinese manufacturing companies. Sustainability. (2022) 14:4520. doi: 10.3390/su14084520

[37] Chen Y Tang G Jin J Xie Q Li J. CEOs’ transformational leadership and product innovation performance: the roles of corporate entrepreneurship and technology orientation. J Prod Innov Manag. (2014) 31:2–17. doi: 10.1111/jpim.12188

[38] Zhao S Xu B Zhang W. Government R&D subsidy policy in China: an empirical examination of effect, priority, and specifics. Technol Forecast Soc Change. (2018) 135:75–82. doi: 10.1016/j.techfore.2017.10.004

[39] Hendricks L. Equipment investment and growth in developing countries. J Dev Econ. (2000) 61:335–64. doi: 10.1016/s0304-3878(00)00060-2

[40] Guo D Qi L Song X. Examining how fiscal policies influence innovation in TCM enterprises: the role of R&D investment and executives with pharmaceutical backgrounds. Front Public Health. (2025) 13:1531622. doi: 10.3389/fpubh.2025.1531622, PMID: 40078775 PMC11897503

[41] Zhang X Tian R Yang Z Zhao C Yao L Lau C . Quality assessment of clinical trial registration with traditional Chinese medicine in WHO registries. BMJ Open. (2019) 9:e025218. doi: 10.1136/bmjopen-2018-025218, PMID: 30782928 PMC6398725

[42] Zhou L Chen Y Liu J Liu Z Gao Y. Evaluating the implementation of evidence-based TCM clinical practice guidelines for cerebral infarction. Eur J Integr Med. (2014) 6:147–55. doi: 10.1016/j.eujim.2014.03.006

[43] Hua H Tang JY Zhao JN Wang T Zhang JH Yu JY . From traditional medicine to modern medicine: the importance of TCM regulatory science (TCMRS) as an emerging discipline. Chin Med. (2025) 20:92. doi: 10.1186/s13020-025-01152-8, PMID: 40563101 PMC12199503

[44] Fu Y. Enterprises’ internationalization, R&D investment and enterprise performance. Finance Res Lett. (2024) 67:105721. doi: 10.1016/j.frl.2024.105721

[45] Da T Liu D. Research and Development expense deduction policy and Enterprise value: an empirical analysis based on A-share manufacturing industry listed companies. Fiscal Sci. (2023) 1:136–53. doi: 10.19477/j.cnki.10-1368/f.2023.01.015

[46] Appelt S Bajgar M Criscuolo C Galindo-Rueda F. R&D tax incentives: evidence on design, incidence and impacts. Organ Econ Co-Oper Dev (OECD). (2016). doi: 10.1787/5jlr8fldqk7j-en

[47] Elschner C Ernst C Licht G Spengel C. What the design of an R&D tax incentive tells about its effectiveness: a simulation of R&D tax incentives in the European Union. J Technol Transf. (2011) 36:233–56. doi: 10.1007/s10961-009-9146-y

[48] Kleer R. Government R&D subsidies as a signal for private investors. Res Policy. (2010) 39:1361–74. doi: 10.1016/j.respol.2010.08.001

[49] Chu X Sun B Mo X Liu J Zhang Y Weng H . Time-series dynamic three-way group decision-making model and its application in TCM efficacy evaluation. Artif Intell Rev. (2023) 56:11095–121. doi: 10.1007/s10462-023-10445-z

[50] Mantila KM Pasmooij AMG Hallgreen CE Mol PGM Van Boven JFM. Medication adherence measurement methods in registration trials supporting the approval of new medicines: a cross-sectional analysis of centralized procedures in the European Union 2010–2020. Clin Pharmacol Ther. (2022) 112:1051–60. doi: 10.1002/cpt.2709, PMID: 35816103 PMC9795959

[51] Singh R Gupta CP Chaudhary P. Defining return on assets (ROA) in empirical corporate finance research. A Crit Rev. (2024). doi: 10.5281/ZENODO.10901886

[52] Li W. Research on the factors of return on equity: empirical analysis in Chinese port industries from 2000-2008 In: Zeng Z Li Y, editors. SPIE proceedings. Singapore, Singapore: SPIE (2012). 834930. 834930–6

[53] Zhang K Zhuang Z. Research on the impact of digital economy policy intensity on stable employment in the manufacturing industry. Jilin Univ Soc Sci J. (2024) 64:155–171+239. doi: 10.15939/j.jujsse.2024.04.jj1

