# Supplementary material for: Research on the impact of traditional Chinese medicine registration innovation-oriented policies and enterprises performance: an empirical analysis based on listed enterprises in China
Source: Front Public Health. 2025 Nov 26;13:1666517. doi: 10.3389/fpubh.2025.1666517 (PMC12689545; doi:10.3389/fpubh.2025.1666517)
Supplement: Supplementary file 1 [file Table_1.DOCX]

Supplement Table Traditional Chinese Medicine Registration Innovation-Oriented Policies List

| **No.** | **Policy Title** | **Issuing agency** | **Year issued** | **Type** |
| --- | --- | --- | --- | --- |
| 1 | Notice on Issuing the Tenth Five-Year Plan for the Traditional Chinese Medicine (TCM) Industry | National Economic and Trade Industry | 2002 | Notifications |
| 2 | Notice on the Outline for the Modernization of Traditional Chinese Medicine (TCM) | Ministry of Science and Technology, State Planning Commission, State Economic and Trade Commission, Ministry of Health, Drug Administration, Intellectual Property Office, Traditional Chinese Medicine Administration, Chinese Academy of Sciences | 2002 | Notifications |
| 3 | Notice on Matters Concerning the Implementation of the Measures for the Administration of Drug Registration (Trial Implementation) | Drug Administration Bureau | 2002 | Notifications |
| 4 | Interpretation of the Measures for the Administration of Drug Registration | Drug Administration Bureau | 2002 | Methods |
| 5 | Measures for the Administration of Drug Registration (Trial Implementation) | Drug Administration Bureau | 2002 | Methods |
| 6 | Notice on Matters Concerning Drug Registration Application and Acceptance | Drug Administration Bureau | 2002 | Notifications |
| 7 | Requirements for Registration Application and Declaration Materials of Traditional Chinese Medicine (TCM) and Natural Medicines | Drug Administration Bureau | 2002 | Rules and Requirements |
| 8 | Notice on Issues Concerning the Restoration of Suspended Approval Numbers for Varieties After the Termination of TCM Variety Protection | Drug Administration Bureau | 2003 | Notifications |
| 9 | Regulations on Traditional Chinese Medicine (TCM) of the People's Republic of China | State Council | 2003 | Ordinances |
| 10 | Notice on Issuing Supplementary Provisions on Drug Registration Management | Drug Administration Bureau | 2003 | Notifications |
| 11 | Notice on Matters Concerning the Replacement of Approval Numbers for TCM Varieties with Suspended Approval Number Effect | State Food and Drug Administration | 2004 | Notifications |
| 12 | Notice on Issuing the Work Plan for the National Special Rectification of Food and Drugs in 2005 | State Council | 2005 | Notifications |
| 13 | Technical Guiding Principles for Pilot-Scale Research of Traditional Chinese Medicine (TCM) and Natural Medicines | National Medical Products Administration Center for Drug Evaluation | 2005 | Guiding principles |
| 14 | Technical Guiding Principles for the Research of Preparations of Traditional Chinese Medicine (TCM) and Natural Medicines | National Medical Products Administration Center for Drug Evaluation | 2005 | Guiding principles |
| 15 | Technical Guiding Principles for the Pretreatment of Raw Materials of Traditional Chinese Medicine (TCM) and Natural Medicines | National Medical Products Administration Center for Drug Evaluation | 2005 | Guiding principles |
| 16 | Technical Guiding Principles for Extraction and Purification Research of Traditional Chinese Medicine (TCM) and Natural Medicines | National Medical Products Administration Center for Drug Evaluation | 2005 | Guiding principles |
| 17 | Notice on Matters Concerning the Implementation of the Measures for the Administration of Drug Registration | State Food and Drug Administration | 2005 | Notifications |
| 18 | Special Examination and Approval Procedures for Drugs of the State Food and Drug Administration | State Food and Drug Administration | 2005 | Rules and Requirements |
| 19 | Measures for the Administration of Drug Registration | State Food and Drug Administration | 2005 | Methods |
| 20 | Interpretation of the Measures for the Administration of Drug Registration | State Food and Drug Administration | 2005 | Methods |
| 21 | Registration Classification and Declaration Materials Requirements for Traditional Chinese Medicine (TCM) and Natural Medicines | State Food and Drug Administration | 2005 | Rules and Requirements |
| 22 | Letter on Issuing Requirements for Registration Declaration Materials of Pharmaceutical Excipients | State Food and Drug Administration | 2005 | Rules and Requirements |
| 23 | Notice on Issuing the Format and Content Writing Requirements and Guiding Principles for the Instructions of Prescription Drugs of Traditional Chinese Medicine (TCM) and Natural Medicines | State Food and Drug Administration | 2006 | Notifications |
| 24 | Notice on Matters Concerning the Protection of Traditional Chinese Medicine (TCM) Varieties | State Food and Drug Administration | 2006 | Notifications |
| 25 | Notice on Strengthening the Registration Management of TCM Containing Poppy Hulls | State Food and Drug Administration | 2006 | Notifications |
| 26 | Notice on Issuing the National Special Action Plan for Rectifying and Standardizing the Drug Market Order | State Council | 2006 | Notifications |
| 27 | Content Writing Requirements for the Instructions of Prescription Drugs of Traditional Chinese Medicine (TCM) and Natural Medicines | State Food and Drug Administration | 2006 | Rules and Requirements |
| 28 | Guiding Principles for Writing the Instructions of Prescription Drugs of Traditional Chinese Medicine (TCM) and Natural Medicines | State Food and Drug Administration | 2006 | Guiding principles |
| 29 | Technical Guiding Principles for Stability Research of Traditional Chinese Medicine (TCM) and Natural Medicines | State Food and Drug Administration | 2006 | Guiding principles |
| 30 | Notice on Issuing the Technical Guiding Principles for Stability Research of Traditional Chinese Medicine (TCM) and Natural Medicines | State Food and Drug Administration | 2006 | Notifications |
| 31 | Notice on Strengthening the Supervision and Inspection of Professional Markets for Chinese Medicinal Materials | State Food and Drug Administration | 2007 | Notifications |
| 32 | Notice on the Early Termination of Protection for Relevant Traditional Chinese Medicine (TCM) Varieties | State Food and Drug Administration | 2007 | Notifications |
| 33 | Notice on Soliciting Opinions on the Supplementary Provisions on TCM Registration Management (Draft for Comment) | State Food and Drug Administration | 2007 | Notifications |
| 34 | Notice of the General Office of the State Council on Further Strengthening Drug Safety Supervision Work | State Council | 2007 | Notifications |
| 35 | Notice on Issuing the Eleventh Five-Year Plan for National Food and Drug Safety | State Council | 2007 | Notifications |
| 36 | Format and Requirements for Clinical Research Summaries of Traditional Chinese Medicine (TCM) and Natural Medicines | National Medical Products Administration Center for Drug Evaluation | 2007 | Rules and Requirements |
| 37 | Format and Requirements for Pharmaceutical Research Summaries of Traditional Chinese Medicine (TCM) and Natural Medicines | National Medical Products Administration Center for Drug Evaluation | 2007 | Rules and Requirements |
| 38 | Format and Requirements for Pharmacological and Toxicological Research Summaries of Traditional Chinese Medicine (TCM) and Natural Medicines | National Medical Products Administration Center for Drug Evaluation | 2007 | Rules and Requirements |
| 39 | Technical Guiding Principles for General Pharmacology Research of Traditional Chinese Medicine (TCM) and Natural Medicines | National Medical Products Administration Center for Drug Evaluation | 2007 | Guiding principles |
| 40 | Principles for Writing Clinical Trial Reports of Traditional Chinese Medicine (TCM) and Natural Medicines | National Medical Products Administration Center for Drug Evaluation | 2007 | Guiding principles |
| 41 | Technical Guiding Principles for Immunotoxicity (Allergy, Photoallergic Reaction) Research of Traditional Chinese Medicine (TCM) and Natural Medicines | National Medical Products Administration Center for Drug Evaluation | 2007 | Guiding principles |
| 42 | Technical Guiding Principles for Local Irritation and Hemolysis Research of Traditional Chinese Medicine (TCM) and Natural Medicines | National Medical Products Administration Center for Drug Evaluation | 2007 | Guiding principles |
| 43 | Technical Guiding Principles for Acute Toxicity Research of Traditional Chinese Medicine (TCM) and Natural Medicines | National Medical Products Administration Center for Drug Evaluation | 2007 | Guiding principles |
| 44 | Basic Technical Requirements for Injections of Traditional Chinese Medicine (TCM) and Natural Medicines | National Medical Products Administration Center for Drug Evaluation | 2007 | Rules and Requirements |
| 45 | Principles for Writing Medical Theory and Literature Materials for Clinical Research Application of Traditional Chinese Medicine (TCM) and Natural Medicines | National Medical Products Administration Center for Drug Evaluation | 2007 | Guiding principles |
| 46 | Principles for Writing Drug Instructions of Traditional Chinese Medicine (TCM) and Natural Medicines | National Medical Products Administration Center for Drug Evaluation | 2007 | Guiding principles |
| 47 | Technical Guiding Principles for Long-Term Toxicity Research of Traditional Chinese Medicine (TCM) and Natural Medicines | National Medical Products Administration Center for Drug Evaluation | 2007 | Guiding principles |
| 48 | Notice on Issuing the Basic Technical Requirements for Injections of Traditional Chinese Medicine (TCM) and Natural Medicines | National Medical Products Administration Center for Drug Evaluation | 2007 | Notifications |
| 49 | Notice on Issuing the Technical Guiding Principles for the Writing Format and Content of Summary Materials of Traditional Chinese Medicine (TCM) and Natural Medicines | National Medical Products Administration Center for Drug Evaluation | 2007 | Notifications |
| 50 | Items of Drug Re-Registration Declaration Materials | National Medical Products Administration Center for Drug Evaluation | 2007 | Rules and Requirements |
| 51 | Matters Concerning Drug Supplementary Registration Applications and Requirements for Declaration Materials | National Medical Products Administration Center for Drug Evaluation | 2007 | Rules and Requirements |
| 52 | Measures for the Administration of Drug Registration | National Medical Products Administration Center for Drug Evaluation | 2007 | Methods |
| 53 | Notice on Matters Concerning the Implementation of the Measures for the Administration of Drug Registration | National Medical Products Administration Center for Drug Evaluation | 2007 | Notifications |
| 54 | Requirements for Declaration Materials of New Traditional Chinese Medicine (TCM) | National Medical Products Administration Center for Drug Evaluation | 2007 | Rules and Requirements |
| 55 | Announcement on Amending the Requirements for Supplementary Application Declaration Materials for Instructions and Labels of Imported Traditional Chinese Medicine (TCM) and Natural Medicines | National Medical Products Administration Center for Drug Evaluation | 2007 | Rules and Requirements |
| 56 | Notice on Issuing Supplementary Provisions on TCM Registration Management | State Food and Drug Administration | 2008 | Notifications |
| 57 | Notice on Issuing 5 Drug Evaluation Technical Standards Including the Handling Principles for TCM Process-Related Issues | State Food and Drug Administration | 2008 | Notifications |
| 58 | Notice on the Outline of the Eleventh Five-Year Plan for the Development of Health Undertakings | State Council | 2008 | Notifications |
| 59 | Notice on Implementing the Food and Drug Safety Project | General Office of the State Council | 2008 | Notifications |
| 60 | Notice on Issuing the Eleventh Five-Year Plan for National Food and Drug Safety | General Office of the State Council | 2008 | Notifications |
| 61 | Regulations on Traditional Chinese Medicine (TCM) of the People's Republic of China | State Council | 2008 | Ordinances |
| 62 | Measures for the Administration of Drug Registration | State Food and Drug Administration | 2008 | Methods |
| 63 | Measures for the Supervision and Administration of Drug Circulation | State Food and Drug Administration | 2008 | Methods |
| 64 | Handling Principles for Issues Related to External Preparations of Traditional Chinese Medicine (TCM) | State Food and Drug Administration | 2008 | Guiding principles |
| 65 | Handling Principles for Issues Related to Traditional Chinese Medicine (TCM) Processes | State Food and Drug Administration | 2008 | Guiding principles |
| 66 | Supplementary Provisions on TCM Registration Management | State Food and Drug Administration | 2008 | Rules and Requirements |
| 67 | Handling Principles for Issues Related to Quality Control Research of Traditional Chinese Medicine (TCM) | State Food and Drug Administration | 2008 | Guiding principles |
| 68 | Judgment Standards and Handling Principles for Unclear Quality Standards of Traditional Chinese Medicine (TCM) | State Food and Drug Administration | 2008 | Guiding principles |
| 69 | Notice on Issuing 5 Drug Evaluation Technical Standards Including the Handling Principles for TCM Process-Related Issues | State Food and Drug Administration | 2008 | Notifications |
| 70 | Notice on Issuing 5 Drug Evaluation Technical Standards Including Chemical Drug Technical Standards | State Food and Drug Administration | 2008 | Notifications |
| 71 | Handling Principles for TCM Varieties Containing Endangered Medicinal Materials | State Food and Drug Administration | 2008 | Guiding principles |
| 72 | Notice on Issuing Supplementary Provisions on TCM Registration Management | State Food and Drug Administration | 2008 | Notifications |
| 73 | Notice on Carrying Out the Re-evaluation of the Safety of TCM Injections | State Food and Drug Administration | 2009 | Notifications |
| 74 | Notice on Issuing the Guiding Principles for the Protection of Traditional Chinese Medicine (TCM) Varieties | State Food and Drug Administration | 2009 | Notifications |
| 75 | Several Opinions on Supporting and Promoting the Development of Traditional Chinese Medicine (TCM) Undertakings | State Council | 2009 | Opinions |
| 76 | Notice on Carrying Out the Re-evaluation of the Safety of TCM Injections | State Food and Drug Administration | 2009 | Notifications |
| 77 | Administrative Provisions on Special Examination and Approval of New Drug Registration | State Food and Drug Administration | 2009 | Rules and Requirements |
| 78 | Notice on Issuing the Plan for the Function Allocation, Internal Institutions and Staffing of the Ministry of Health and the State Administration of Traditional Chinese Medicine (TCM) | General Office of the State Council | 2010 | Notifications |
| 79 | Notice on Issuing the Plan for the Function Allocation, Internal Institutions and Staffing of the State Pharmaceutical Administration | General Office of the State Council | 2010 | Notifications |
| 80 | Notice on Issuing the Provisions on the Function Allocation, Internal Institutions and Staffing of the State Bureau of Quality and Technical Supervision | General Office of the State Council | 2010 | Notifications |
| 81 | Notice on Issuing the Guiding Opinions on Establishing and Standardizing the Essential Drug Procurement Mechanism for Government-Run Primary Medical and Health Institutions | General Office of the State Council | 2010 | Notifications |
| 82 | Notice on Issuing the Guidelines for the Construction of TCM Culture in TCM Hospitals | National Administration of Traditional Chinese Medicine | 2010 | Notifications |
| 83 | Notice on Issuing the Provisions on the Administration of Drug Technology Transfer Registration | State Food and Drug Administration | 2010 | Notifications |
| 84 | Technical Requirements for the Rationality of Dosage Form Selection for Modified-Dosage-Form TCM Varieties | State Food and Drug Administration | 2010 | Rules and Requirements |
| 85 | Notice on Doing a Good Job in the Re-evaluation of the Safety of TCM Injections in 2010 | State Food and Drug Administration | 2010 | Notifications |
| 86 | Notice on Further Strengthening the Supervision and Inspection of TCM Production | State Food and Drug Administration | 2010 | Notifications |
| 87 | Notice on Issuing the Technical Guiding Principles for the Change Research of Marketed TCM (I) | State Food and Drug Administration | 2011 | Notifications |
| 88 | Notice on Issuing the Interpretation of the Technical Guiding Principles for the Change Research of Marketed TCM (I) | State Food and Drug Administration | 2011 | Notifications |
| 89 | Notice on Issuing the Technical Guiding Principles for Clinical Research of Traditional Chinese Medicine (TCM) and Natural Medicines in the Treatment of Coronary Heart Disease Angina Pectoris and Female Menopausal Syndrome | State Food and Drug Administration | 2011 | Notifications |
| 90 | Technical Guiding Principles for the Change Research of Marketed Traditional Chinese Medicine (TCM) (I) | State Food and Drug Administration | 2011 | Guiding principles |
| 91 | Measures for the Management of Drug Adverse Reaction Reporting and Monitoring | Ministry of Health | 2011 | Methods |
| 92 | Notice on Issuing the Provisions on the Main Responsibilities, Internal Institutions and Staffing of the State Food and Drug Administration | State Council | 2012 | Notifications |
| 93 | Notice on Revising and Issuing the Interim Measures for the Administration of Licensed Pharmacist Registration | State Food and Drug Administration | 2012 | Notifications |
| 94 | Notice on Issuing the Twelfth Five-Year Plan for National Drug Safety | State Council | 2012 | Notifications |
| 95 | Notice on Issuing the Plan and Implementation Plan for Deepening the Reform of the Medical and Health System During the Twelfth Five-Year Plan Period | State Council | 2012 | Notifications |
| 96 | Notice on Issuing the Plan and Implementation Plan for Deepening the Reform of the Medical and Health System During the Twelfth Five-Year Plan Period | State Council | 2012 | Notifications |
| 97 | Notice on Issuing the 2012 Action Plan for Implementing the Outline for Quality Development | General Office of the State Council | 2012 | Notifications |
| 98 | Notice on Issuing the Twelfth Five-Year Plan for the Development of Domestic Trade | General Office of the State Council | 2012 | Notifications |
| 99 | Notice on Issuing the Medium- and Long-Term Development Plan Outline for Traditional Chinese Medicine (TCM) Standardization (2011-2020) | National Administration of Traditional Chinese Medicine | 2012 | Notifications |
| 100 | Notice on Strengthening the Management Report on the Export of Chinese Medicinal Materials | General Office of the State Council | 2013 | Notifications |
| 101 | Opinions on Consolidating and Improving the Essential Drug System and the New Primary-Level Operation Mechanism | General Office of the State Council | 2013 | Opinions |
| 102 | Notice on Doing a Good Job in Matters Related to Drug Technology Transfer During the Implementation of the Revised Good Manufacturing Practice (GMP) for Drugs | State Food and Drug Administration | 2013 | Notifications |
| 103 | Drug Administration Law of the People's Republic of China | Standing Committee of the National People's Congress | 2013 | Laws |
| 104 | Announcement on Issuing the Technical Guiding Principles for the Study of Dosage Form Change of Traditional Chinese Medicine (TCM) and Natural Medicines | State Food and Drug Administration | 2014 | Announcements |
| 105 | Guiding Opinions on Accelerating the Construction of Population Health Informatization | The Health and Family Planning Commission, the National Administration of Traditional Chinese Medicine | 2014 | Opinions |
| 106 | Regulations on the Administration of Narcotic Drugs and Psychotropic Substances | State Council | 2014 | Ordinances |
| 107 | Technical Guiding Principles for the Study of Dosage Form Change of Traditional Chinese Medicine (TCM) and Natural Medicines | State Food and Drug Administration | 2014 | Guiding principles |
| 108 | Notice on the Early Termination of Protection for Relevant Traditional Chinese Medicine (TCM) Varieties | State Food and Drug Administration | 2015 | Notifications |
| 109 | Inspection and Acceptance Measures for Good Manufacturing Practice (GMP) for Traditional Chinese Medicine (TCM) Production | Ministry of Agriculture | 2015 | Methods |
| 110 | Guiding Opinions on Improving the Centralized Drug Procurement Work in Public Hospitals | General Office of the State Council | 2015 | Opinions |
| 111 | Notice on Forwarding the Protection and Development Plan for Chinese Medicinal Materials (2015-2020) of the Ministry of Industry and Information Technology and Other Departments | General Office of the State Council | 2015 | Notifications |
| 112 | Protection and Development Plan for Chinese Medicinal Materials (2015-2020) of the Ministry of Industry and Information Technology and Other Departments | State Council | 2015 | Notifications |
| 113 | Notice on Issuing the Development Plan for Traditional Chinese Medicine (TCM) Health Services (2015-2020) | State Council | 2015 | Notifications |
| 114 | Development Plan for Traditional Chinese Medicine (TCM) Health Services (2015-2020) | General Office of the State Council | 2015 | Notifications |
| 115 | Overall Goals of the Development Plan for Traditional Chinese Medicine (TCM) Health Services (2015-2020) | State Council | 2015 | Guiding principles |
| 116 | Key Contents of the Development Plan for Traditional Chinese Medicine (TCM) Health Services (2015-2020) | State Council | 2015 | Guiding principles |
| 117 | Opinions on Reforming the Examination and Approval System for Drugs and Medical Devices | State Council | 2015 | Opinions |
| 118 | General Principles for Clinical Research of New Traditional Chinese Medicine (TCM) | State Food and Drug Administration | 2015 | Guiding principles |
| 119 | Technical Guiding Principles for Irradiation Sterilization of Traditional Chinese Medicine (TCM) | State Food and Drug Administration | 2015 | Guiding principles |
| 120 | Opinions on Reforming the Examination and Approval System for Drugs and Medical Devices | State Council | 2015 | Opinions |
| 121 | Drug Administration Law of the People's Republic of China | Standing Committee of the National People's Congress | 2015 | Laws |
| 122 | Suggestions on Strengthening Supply-Side Structural Reform to Promote the Innovative Development of Traditional Chinese Medicine (TCM) | Standing Committee of the National People's Congress | 2016 | Opinions |
| 123 | Suggestions on Giving Priority to New Traditional Chinese Medicine (TCM) to Enter the Green Channel of the National Medical Insurance Catalog | Standing Committee of the National People's Congress | 2016 | Opinions |
| 124 | Notice on the Outline for the Modernization Development of Traditional Chinese Medicine (TCM) | General Office of the State Council | 2016 | Notifications |
| 125 | Notice on the Seventh Five-Year Plan (2016-2020) for Carrying Out Legal Popularization and Education in the Traditional Chinese Medicine (TCM) Industry | National Administration of Traditional Chinese Medicine | 2016 | Notifications |
| 126 | Issuance of the "Healthy China 2030" Plan Outline | State Council | 2016 | Notifications |
| 127 | Deepen Cooperation Between the Two Regions and Jointly Promote the Modernization and Internationalization of Traditional Chinese Medicine (TCM) | Ministry of Science and Technology | 2016 | Notifications |
| 128 | Development Plan Guide for the Pharmaceutical Industry | The National Health and Family Planning Commission learned that the Ministry of Industry and Information Technology and the National Health and Family Planning Commission | 2016 | Notifications |
| 129 | Measures for the Quarantine Supervision and Administration of Inbound and Outbound Chinese Medicinal Materials | General Administration of Quality Supervision | 2016 | Methods |
| 130 | Legislative Ideas and Highlights of the "Traditional Chinese Medicine (TCM) Law" | National Administration of Traditional Chinese Medicine | 2016 | Guiding principles |
| 131 | Traditional Chinese Medicine (TCM) Law of the People's Republic of China | Standing Committee of the National People's Congress | 2016 | Laws |
| 132 | White Paper on "Traditional Chinese Medicine (TCM) in China" | State Council | 2016 | Notifications |
| 133 | Notice on Issuing the Comprehensive Prevention and Treatment Work Plan for Stroke | National Administration of Traditional Chinese Medicine | 2016 | Notifications |
| 134 | Implementation Regulations of the Drug Administration Law of the People's Republic of China | State Council | 2016 | Ordinances |
| 135 | "Foreign Traditional Chinese Medicine (TCM)" Become a "New Favorite" for Chinese People to Purchase | National Administration of Traditional Chinese Medicine | 2016 | Guiding principles |
| 136 | The Development Path of Traditional Chinese Medicine (TCM) in the Next 15 Years | National Administration of Traditional Chinese Medicine | 2016 | Guiding principles |
| 137 | Notice of the State Council on Issuing the Strategic Planning Outline for the Development of Traditional Chinese Medicine (TCM) (2016-2030) | State Council | 2016 | Notifications |
| 138 | Strategic Planning Outline for the Development of Traditional Chinese Medicine (TCM) (2016-2030) | State Council | 2016 | Notifications |
| 139 | Guiding Opinions on Promoting the Healthy Development of the Pharmaceutical Industry | State Council | 2016 | Opinions |
| 140 | Thirteenth Five-Year Plan for the Development of Traditional Chinese Medicine (TCM) | National Administration of Traditional Chinese Medicine | 2016 | Rules and Requirements |
| 141 | Notice on Carrying Out the Establishment of National Traditional Chinese Medicine (TCM) Health Tourism Demonstration Zones (Bases, Projects) | National Tourism Administration, National Administration of Traditional Chinese Medicine | 2016 | Notifications |
| 142 | Implementation Regulations of the Drug Administration Law of the People's Republic of China | State Council | 2016 | Ordinances |
| 143 | Announcement on Issuing 5 Clinical Research Technical Guiding Principles Including the Technical Guiding Principles for Clinical Research of New Traditional Chinese Medicine (TCM) for Irritable Bowel Syndrome | National Medical Products Administration | 2017 | Guiding principles |
| 144 | Issuance of the "Opinions on Deepening the Reform of the Examination and Approval System to Encourage Innovation in Drugs and Medical Devices" | State Council | 2017 | Opinions |
| 145 | Notice on Issuing the Implementation Plan for the "Traditional Chinese Medicine (TCM) in China" Campaign - TCM Health Culture Promotion Initiative (2016-2020) | National Administration of Traditional Chinese Medicine | 2017 | Notifications |
| 146 | Notice on Issuing the Interim Measures for the Fund Management of the "Baiqianwan" Talent Project (Qihuang Project) for the Inheritance and Innovation of Traditional Chinese Medicine (TCM) and the Interim Measures for the Fund Management of the Fourth National Survey of TCM Resources | National Administration of Traditional Chinese Medicine | 2017 | Notifications |
| 147 | Notice of the State Council on Issuing the Thirteenth Five-Year Plan for Deepening the Reform of the Medical and Health System | State Council | 2017 | Notifications |
| 148 | Notice of the Two Departments on Studying, Publicizing and Implementing the "Traditional Chinese Medicine (TCM) Law of the People's Republic of China" | National Health and Family Planning Commission, National Administration of Traditional Chinese Medicine | 2017 | Notifications |
| 149 | Several Opinions on Further Reforming and Improving Policies on the Production, Circulation and Use of Drugs | State Council | 2017 | Opinions |
| 150 | Notice on Issuing the Thirteenth Five-Year National Food Safety Plan and the Thirteenth Five-Year National Drug Safety Plan | State Council | 2017 | Notifications |
| 151 | Notice on Issuing the Key Work Tasks for Deepening the Reform of the Medical and Health System in 2017 | State Council | 2017 | Notifications |
| 152 | Notice of the General Office of the State Council on Issuing the Key Work Tasks for Deepening the Reform of the Medical and Health System in 2017 | General Office of the State Council | 2017 | Notifications |
| 153 | Guiding Opinions on Comprehensively Promoting Scientific and Technological Innovation in Health and Sanitation | The Department of Science and Technology of the Ministry of Health and Family Planning, the Food and Drug Administration, and the Bureau of Traditional Chinese Medicine | 2017 | Opinions |
| 154 | Issuance of the "Opinions on Deepening the Reform of the Examination and Approval System to Encourage Innovation in Drugs and Medical Devices" | Military Commission's Logistics Support Department's Health Bureau | 2017 | Opinions |
| 155 | Acceptance and Examination Guide for Registration and Approval of Traditional Chinese Medicine (TCM) and Natural Medicines | National Medical Products Administration | 2017 | Guiding principles |
| 156 | Technical Guiding Principles for the Evaluation of Traditional Chinese Medicine (TCM) Resources | National Medical Products Administration Center for Drug Evaluation | 2017 | Guiding principles |
| 157 | Explanation on the "Technical Guiding Principles for the Naming of Generic Names of Chinese Patent Medicines" | National Medical Products Administration Center for Drug Evaluation | 2017 | Guiding principles |
| 158 | Technical Guiding Principles for the Study of Production Process Changes of Marketed Traditional Chinese Medicine (TCM) | National Medical Products Administration Center for Drug Evaluation | 2017 | Guiding principles |
| 159 | Traditional Chinese Medicine (TCM) Law of the People's Republic of China | National Administration of Traditional Chinese Medicine | 2017 | Laws |
| 160 | The State Council Amends Some Provisions of Administrative Regulations Including the "Regulations on the Protection of Traditional Chinese Medicine (TCM) Varieties" | State Council | 2018 | Ordinances |
| 161 | Announcement on Issuing the Technical Guiding Principles for Clinical Research of Syndrome-Based New Traditional Chinese Medicine (TCM) | National Medical Products Administration | 2018 | Announcements |
| 162 | Basis for Formulating the "Technical Guiding Principles for Clinical Research of Syndrome-Based New Traditional Chinese Medicine (TCM)" | National Medical Products Administration | 2018 | Guiding principles |
| 163 | Scope of Application of the "Technical Guiding Principles for Clinical Research of Syndrome-Based New Traditional Chinese Medicine (TCM)" | National Medical Products Administration | 2018 | Guiding principles |
| 164 | Several Opinions on Strengthening the Work of Ethnic Minority Medicine in the New Era | National Health Commission, Ministry of Civil Affairs, National Development and Reform Commission, Ministry of Education, Ministry of Science and Technology, Ministry of Finance | 2018 | Opinions |
| 165 | Notice on Implementing the Requirements of the State Council's "Separation of Certificates and Licenses" Reform to Do a Good Job in Drug Supervision-Related Approval Work | State Food and Drug Administration | 2018 | Notifications |
| 166 | Notice on Further Strengthening the Supervision of Drugs, Medical Devices and Cosmetics During Institutional Reform | State Food and Drug Administration | 2018 | Notifications |
| 167 | Notice on Carrying Out the "High-Quality Service at the Grassroots Level" Activity | Health and Wellness Commission, the National Administration of Traditional Chinese Medicine | 2018 | Notifications |
| 168 | Guiding Opinions on Deepening the Mentorship Education of Traditional Chinese Medicine (TCM) | National Administration of Traditional Chinese Medicine | 2018 | Opinions |
| 169 | Several Opinions on Strengthening the Work of Ethnic Minority Medicine in the New Era | National Health Commission, Ministry of Civil Affairs, National Development and Reform Commission, Ministry of Education, Ministry of Science and Technology, Ministry of Finance, Ministry of Human Resources and Social Security, Ministry of Commerce, Ministry of Culture and Tourism, National Health Commission, Medical Insurance Bureau, Drug Administration Bureau, Intellectual Property Administration Bureau | 2018 | Opinions |
| 170 | Notice on Clarifying the Management System and Other Work After the End of the Pilot Project of the Circulation Traceability System for Meat, Vegetables and Chinese Medicinal Materials | Ministry of Commerce General Office Ministry of Finance General Office | 2018 | Notifications |
| 171 | Notice on Issuing the "Baiqianwan" Talent Project (Qihuang Project) for the Inheritance and Innovation of Traditional Chinese Medicine (TCM) - National TCM Leading Talent Support Plan | National Administration of Traditional Chinese Medicine | 2018 | Notifications |
| 172 | Notice on Doing a Good Job in the Filing of Traditional Chinese Medicine (TCM) Preparations Prepared by Medical Institutions Using Traditional Processes | State Food and Drug Administration | 2018 | Notifications |
| 173 | Notice on Issuing the National Planning for the Construction of Genuine Medicinal Materials Production Bases (2018-2025) | Ministry of Agriculture and Rural Affairs, Food and Drug Administration, Bureau of Traditional Chinese Medicine | 2018 | Notifications |
| 174 | Decision of the State Food and Drug Administration on Amending Some Rules | National Medical Products Administration | 2018 | Methods |
| 175 | In Response to Defects Such as Unclear Drug Content and Unknown Side Effects, the State Food and Drug Administration Issues Guiding Principles | National Medical Products Administration | 2018 | Guiding principles |
| 176 | Guiding Opinions of the State Administration of Traditional Chinese Medicine (TCM) on Promoting the Integrated Development of TCM Health Services and the Internet | National Administration of Traditional Chinese Medicine | 2018 | Opinions |
| 177 | Notice on Issuing the Action Plan for Further Improving Medical Services (2018-2020) | The Health and Family Planning Commission, the National Administration of Traditional Chinese Medicine | 2018 | Notifications |
| 178 | Technical Guiding Principles for Clinical Research of Syndrome-Based New Traditional Chinese Medicine (TCM) | National Medical Products Administration | 2018 | Guiding principles |
| 179 | Drafting Instructions for the "Technical Guiding Principles for Clinical Research of Syndrome-Based New Traditional Chinese Medicine (TCM)" | National Medical Products Administration | 2018 | Guiding principles |
| 180 | Announcement on Adjusting the Examination and Approval Procedures for Drug Clinical Trials | National Medical Products Administration | 2018 | Announcements |
| 181 | Administrative Provisions on Simplified Registration and Approval of Traditional Chinese Medicine (TCM) Compound Preparations of Ancient Classic Prescriptions | National Medical Products Administration | 2018 | Rules and Requirements |
| 182 | Announcement of the National Medical Products Administration (NMPA) on Issuing the Administrative Provisions on Simplified Registration and Approval of Traditional Chinese Medicine (TCM) Compound Preparations of Ancient Classic Prescriptions | National Medical Products Administration | 2018 | Announcements |
| 183 | Announcement on Protected Traditional Chinese Medicine (TCM) Varieties | National Medical Products Administration | 2019 | Announcements |
| 184 | Announcement of the National Medical Products Administration (NMPA) on Protected Traditional Chinese Medicine (TCM) Varieties (No. 2 on Extending Protection Period) | National Medical Products Administration | 2019 | Announcements |
| 185 | National Medical Products Administration (NMPA) | National Medical Products Administration | 2019 | Announcements |
| 186 | Announcement on Matters Concerning Further Improving the Associated Examination and Approval and Supervision of Drugs | National Medical Products Administration | 2019 | Announcements |
| 187 | Inheriting the Essence and Innovating While Upholding Tradition - Four Key Points of the "Opinions of the CPC Central Committee and the State Council on Promoting the Inheritance and Innovative Development of Traditional Chinese Medicine (TCM)" | State Council | 2019 | Opinions |
| 188 | Opinions on Promoting the Inheritance and Innovative Development of Traditional Chinese Medicine (TCM) | State Council | 2019 | Opinions |
| 189 | Implementation Regulations of the Drug Administration Law of the People's Republic of China | Standing Committee of the National People's Congress | 2019 | Ordinances |
| 190 | Notice on Issuing the National Planning for the Construction of Genuine Medicinal Materials Production Bases (2018-2025) | Ministry of Agriculture and Rural Affairs, Food and Drug Administration, Bureau of Traditional Chinese Medicine | 2019 | Notifications |
| 191 | Drug Administration Law of the People's Republic of China | Standing Committee of the National People's Congress | 2019 | Laws |
| 192 | Opinions on Promoting the Development of Forest Health and Wellness Industry | Forest and Grass Bureau, Ministry of Civil Affairs, National Health Commission, Bureau of Traditional Chinese Medicine | 2019 | Opinions |
| 193 | Drug Administration Law of the People's Republic of China | National Medical Products Administration | 2019 | Laws |
| 194 | Requirements for Declaration Materials of Substance Benchmarks for Traditional Chinese Medicine (TCM) Compound Preparations of Ancient Classic Prescriptions | National Medical Products Administration Center for Drug Evaluation | 2019 | Rules and Requirements |
| 195 | Requirements for Declaration Materials of Traditional Chinese Medicine (TCM) Compound Preparations of Ancient Classic Prescriptions | National Medical Products Administration Center for Drug Evaluation | 2019 | Rules and Requirements |
| 196 | Interpretation of the Opinions on Promoting the Inheritance and Innovative Development of Traditional Chinese Medicine (TCM) | State Council | 2019 | Opinions |
| 197 | Interpretation of the Implementation Regulations of the Drug Administration Law of the People's Republic of China | State Council | 2019 | Ordinances |
| 198 | Implementation Opinions on Promoting the Inheritance and Innovative Development of Traditional Chinese Medicine (TCM) | National Medical Products Administration | 2020 | Opinions |
| 199 | Announcement on Issuing the "Registration Classification and Declaration Materials Requirements for Traditional Chinese Medicine (TCM)" | National Medical Products Administration | 2020 | Announcements |
| 200 | What Are the Changes in the Registration Classification and Declaration Materials Requirements for Traditional Chinese Medicine (TCM) Compared with the Previous Ones? | National Medical Products Administration Center for Drug Evaluation | 2020 | Guiding principles |
| 201 | Opinions on the "Verification Principles for Key Information of Ancient Classic Prescriptions (Draft for Comment)" and "Key Information Table of Ancient Classic Prescriptions (7 Prescriptions) (Draft for Comment)" | National Medical Products Administration, National Administration of Traditional Chinese Medicine | 2020 | Opinions |
| 202 | Opinions on 6 Documents Including the "Special Provisions on Traditional Chinese Medicine (TCM) Registration Management (Draft for Comment)" | National Medical Products Administration | 2020 | Opinions |
| 203 | Announcement on Issuing 3 Guiding Principles Including the "Technical Guiding Principles for Quality Control Research of Medicinal Materials Used in New Traditional Chinese Medicine (TCM) (Trial Implementation)" | National Medical Products Administration | 2020 | Announcements |
| 204 | Announcement on Issuing the "Technical Guiding Principles for Production Process Research of Traditional Chinese Medicine (TCM) Compound Preparations (Trial Implementation)" | National Medical Products Administration | 2020 | Announcements |
| 205 | Launch of the Pilot Work of the "Work Plan for the Innovative Development of Drug and Medical Device Supervision in the Guangdong-Hong Kong-Macao Greater Bay Area" | National Medical Products Administration | 2020 | Guiding principles |
| 206 | Major Innovative Measures of the "Work Plan for the Innovative Development of Drug and Medical Device Supervision in the Guangdong-Hong Kong-Macao Greater Bay Area" | National Medical Products Administration | 2020 | Guiding principles |
| 207 | Implementation Opinions on Deepening the Collaboration Between Medical and Educational Sectors to Further Promote the Reform and High-Quality Development of Traditional Chinese Medicine (TCM) Education | Ministry of Education, National Health Commission, National Administration of Traditional Chinese Medicine | 2020 | Opinions |
| 208 | Focused Perspective on the Progress of Science and Technology in Epidemic Prevention and Control | National Medical Products Administration | 2020 | Guiding principles |
| 209 | Notice on Issuing the Healthy China Initiative - Cancer Prevention and Control Implementation Plan (2019-2022) | State Council | 2020 | Notifications |
| 210 | The Revised "Measures for the Administration of Drug Registration" Encourages the Inheritance and Innovation of Traditional Chinese Medicine (TCM) | National Medical Products Administration | 2020 | Guiding principles |
| 211 | Relevant Documents and Work of the Revised "Measures for the Administration of Drug Registration" | National Medical Products Administration | 2020 | Guiding principles |
| 212 | Measures for the Supervision and Administration of Drug Production | The State Administration for Market Regulation | 2020 | Methods |
| 213 | Interpretation of the Measures for the Administration of Drug Registration | The State Administration for Market Regulation | 2020 | Methods |
| 214 | Measures for the Administration of Drug Registration | National Medical Products Administration | 2020 | Methods |
| 215 | Notice of Measures for the Supervision and Administration of Drug Production | National Medical Products Administration | 2020 | Methods |
| 216 | Announcement on Issuing the Good Clinical Practice (GCP) for Drugs | National Medical Products Administration | 2020 | Announcements |
| 217 | Announcement on Matters Concerning the Implementation of the 2020 Edition of the "Pharmacopoeia of the People's Republic of China" | National Medical Products Administration | 2020 | Announcements |
| 218 | Issuance of Regulations on the Development of Traditional Chinese Medicine (TCM) | National Medical Products Administration | 2020 | Ordinances |
| 219 | Evaluation Procedures and Relevant Provisions for Traditional Chinese Medicine (TCM) Compound Preparations of Ancient Classic Prescriptions | National Medical Products Administration | 2020 | Guiding principles |
| 220 | Situations Where Changes in Production Processes of Marketed Traditional Chinese Medicine (TCM) Need to Be Declared as Improved New TCM | National Medical Products Administration | 2020 | Guiding principles |
| 221 | Scope of Traditional Chinese Medicine (TCM) Compound Preparations of Ancient Classic Prescriptions | National Medical Products Administration | 2020 | Guiding principles |
| 222 | What Is the Difference Between Generic Drugs with the Same Name and Same Formula and Generic Drugs in the Original Registration Classification? | National Medical Products Administration | 2020 | Guiding principles |
| 223 | Requirements for Submitting Declaration Materials for Traditional Chinese Medicine (TCM) and Natural Medicines Marketed Overseas but Not in China | National Medical Products Administration | 2020 | Guiding principles |
| 224 | Background of the Revision of Traditional Chinese Medicine (TCM) Registration Classification | National Medical Products Administration | 2020 | Guiding principles |
| 225 | Concept of the Revision of Traditional Chinese Medicine (TCM) Registration Classification | National Medical Products Administration | 2020 | Guiding principles |
| 226 | Implementation Opinions on Promoting the Inheritance and Innovative Development of Traditional Chinese Medicine (TCM) | National Medical Products Administration | 2020 | Opinions |
| 227 | Traditional Chinese Medicine (TCM) Registration Acceptance and Examination Guide (Draft for Comment) | National Medical Products Administration | 2020 | Opinions |
| 228 | Technical Guiding Principles for Homogenization Research of Traditional Chinese Medicine (TCM) (Trial Implementation) | National Medical Products Administration | 2020 | Guiding principles |
| 229 | Technical Guiding Principles for Production Process Research of Traditional Chinese Medicine (TCM) Compound Preparations (Trial Implementation) | National Medical Products Administration | 2020 | Guiding principles |
| 230 | Technical Guiding Principles for Sample Research for Toxicological Research of New Traditional Chinese Medicine (TCM) (Trial Implementation) | National Medical Products Administration | 2020 | Guiding principles |
| 231 | Drafting Instructions for the "Technical Guiding Principles for Sample Research for Toxicological Research of New Traditional Chinese Medicine (TCM) (Trial Implementation)" | National Medical Products Administration | 2020 | Guiding principles |
| 232 | Technical Guiding Principles for Quality Control Research of Medicinal Materials Used in New Traditional Chinese Medicine (TCM) (Trial Implementation) | National Medical Products Administration | 2020 | Guiding principles |
| 233 | Technical Guiding Principles for Pharmaceutical Research at Various Stages of New Traditional Chinese Medicine (TCM) Research (Trial Implementation) | National Medical Products Administration | 2020 | Guiding principles |
| 234 | Pharmaceutical Data Requirements for Communication Meetings During the Research of New Traditional Chinese Medicine (TCM) (Trial Implementation) | National Medical Products Administration | 2020 | Rules and Requirements |
| 235 | Technical Guiding Principles for Quality Standard Research of New Traditional Chinese Medicine (TCM) (Trial Implementation) | National Medical Products Administration | 2020 | Guiding principles |
| 236 | Technical Guiding Principles for Biological Effect Detection Research of Traditional Chinese Medicine (TCM) (Trial Implementation) | National Medical Products Administration | 2020 | Guiding principles |
| 237 | Announcement on Issuing the "Registration Classification and Declaration Materials Requirements for Traditional Chinese Medicine (TCM)" | National Medical Products Administration | 2020 | Announcements |
| 238 | Announcement on Matters Concerning the Implementation of the "Measures for the Administration of Drug Registration" | National Medical Products Administration | 2020 | Notifications |
| 239 | Announcement on Issuing the "Technical Guiding Principles for Sample Research for Toxicological Research of New Traditional Chinese Medicine (TCM) (Trial Implementation)" | National Medical Products Administration Center for Drug Evaluation | 2020 | Announcements |
| 240 | Announcement on Issuing the "Technical Guiding Principles for Pharmaceutical Research at Various Stages of New Traditional Chinese Medicine (TCM) Research (Trial Implementation)" | National Medical Products Administration Center for Drug Evaluation | 2020 | Announcements |
| 241 | Measures for the Administration of Drug Registration | State Administration for Market Regulation | 2020 | Methods |
| 242 | Registration Classification and Declaration Materials Requirements for Traditional Chinese Medicine (TCM) | National Medical Products Administration | 2020 | Rules and Requirements |
| 243 | Announcement on Issuing the "Registration Classification and Declaration Materials Requirements for Traditional Chinese Medicine (TCM)" | National Medical Products Administration | 2020 | Announcements |
| 244 | Measures for the Administration of Drug Registration | State Administration for Market Regulation | 2020 | Methods |
| 245 | Notice of the General Office of the National Medical Products Administration (NMPA) on the Early Termination of Protection for Relevant Traditional Chinese Medicine (TCM) Varieties | National Medical Products Administration | 2021 | Notifications |
| 246 | Announcement on Issuing the "Implementation Measures for the Early Resolution Mechanism of Drug Patent Disputes (Trial Implementation)" | National Medical Products Administration, National Intellectual Property Administration | 2021 | Announcements |
| 247 | Notice on Standardizing the Clinical Use of Traditional Chinese Medicine (TCM) Dispensing Granules in Medical Institutions | Office of the Health and Wellness Commission, Office of the Commission of Traditional Chinese Medicine | 2021 | Notifications |
| 248 | Background of the Drafting of the "Implementation Opinions of the National Medical Products Administration (NMPA) on Promoting the Inheritance and Innovative Development of Traditional Chinese Medicine (TCM)" | National Medical Products Administration | 2021 | Guiding principles |
| 249 | Overall Ideas and Goals of the Drafting of the "Implementation Opinions of the National Medical Products Administration (NMPA) on Promoting the Inheritance and Innovative Development of Traditional Chinese Medicine (TCM)" | National Medical Products Administration | 2021 | Guiding principles |
| 250 | Measures in the "Implementation Opinions of the National Medical Products Administration (NMPA) on Promoting the Inheritance and Innovative Development of Traditional Chinese Medicine (TCM)" for Encouraging TCM Innovation | National Medical Products Administration | 2021 | Guiding principles |
| 251 | Main Contents of the "Implementation Opinions of the National Medical Products Administration (NMPA) on Promoting the Inheritance and Innovative Development of Traditional Chinese Medicine (TCM)" | National Medical Products Administration | 2021 | Guiding principles |
| 252 | Measures in the "Implementation Opinions of the National Medical Products Administration (NMPA) on Promoting the Inheritance and Innovative Development of Traditional Chinese Medicine (TCM)" for Strengthening TCM Supervision | National Medical Products Administration | 2021 | Guiding principles |
| 253 | Ensuring the Quality of Traditional Chinese Medicine (TCM) Dispensing Granules | National Medical Products Administration | 2021 | Guiding principles |
| 254 | Notice on Several Policy Measures for Accelerating the Characteristic Development of Traditional Chinese Medicine (TCM) | State Council | 2021 | Notifications |
| 255 | Several Policy Measures for Accelerating the Characteristic Development of Traditional Chinese Medicine (TCM) | General Office of the State Council | 2021 | Notifications |
| 256 | Traditional Chinese Medicine (TCM) Undertakings During the "14th Five-Year Plan" Period | National Medical Products Administration | 2021 | Guiding principles |
| 257 | Announcement on Issuing the "Technical Guiding Principles for Pharmaceutical Change Research of Marketed Traditional Chinese Medicine (TCM) (Trial Implementation)" | National Medical Products Administration | 2021 | Announcements |
| 258 | Issuance of the "Implementation Opinions on Comprehensively Strengthening the Construction of Drug Supervision Capabilities" | State Council | 2021 | Opinions |
| 259 | Issuance of a Notice to Support the Export of National Traditional Chinese Medicine (TCM) Services | Ministry of Commerce, National Administration of Traditional Chinese Medicine | 2021 | Notifications |
| 260 | Implementation Opinions on Comprehensively Strengthening the Construction of Drug Supervision Capabilities | State Council | 2021 | Opinions |
| 261 | Joint Issuance of a Notice: Supporting the Development of National Traditional Chinese Medicine (TCM) Service Export Bases | Ministry of Commerce, National Administration of Traditional Chinese Medicine | 2021 | Notifications |
| 262 | Opinions on Further Strengthening the Work of Traditional Chinese Medicine (TCM) in General Hospitals and Promoting the Collaborative Development of Traditional Chinese and Western Medicine | Health and Wellness Commission | 2021 | Opinions |
| 263 | Announcement on Issuing the "Good Pharmacovigilance Practice (GVP)" | National Medical Products Administration | 2021 | Announcements |
| 264 | Issuance of the "Guiding Opinions on Deepening the Reform of the Title System for Health Professional and Technical Personnel" | The Professional and Technical Department of the Ministry of Human Resources and Social Security, the Personnel Department of the National Health Commission, and the Personnel and Education Department of the National Administration of Traditional Chinese Medicine | 2021 | Opinions |
| 265 | Guiding Opinions on Deepening the Reform of the Title System for Health Professional and Technical Personnel | Ministry of Human Resources and Social Security, Ministry of Health and Family Planning, Bureau of Traditional Chinese Medicine | 2021 | Opinions |
| 266 | Guiding Principles for Writing Declaration Materials on Traditional Chinese Medicine (TCM) Theory of New TCM Compound Preparations (Trial Implementation) | National Administration of Traditional Chinese Medicine | 2021 | Guiding principles |
| 267 | Guiding Principles for Writing Instructions of Traditional Chinese Medicine (TCM) Compound Preparations of Ancient Classic Prescriptions (Trial Implementation) | National Administration of Traditional Chinese Medicine | 2021 | Guiding principles |
| 268 | Announcement on Issuing the "Guiding Principles for Writing Declaration Materials on Traditional Chinese Medicine (TCM) Theory of New TCM Compound Preparations (Trial Implementation)" and "Guiding Principles for Writing Instructions of TCM Compound Preparations of Ancient Classic Prescriptions (Trial Implementation)" | National Medical Products Administration Center for Drug Evaluation | 2021 | Announcements |
| 269 | Guiding Principles for Writing Declaration Materials on Traditional Chinese Medicine (TCM) Theory of New TCM Compound Preparations (Trial Implementation) | National Administration of Traditional Chinese Medicine | 2021 | Guiding principles |
| 270 | Technical Guiding Principles for Quality Research of New Traditional Chinese Medicine (TCM) (Trial Implementation) | National Administration of Traditional Chinese Medicine | 2021 | Guiding principles |
| 271 | Guiding Principles for Writing Instructions of Traditional Chinese Medicine (TCM) Compound Preparations of Ancient Classic Prescriptions (Trial Implementation) | National Administration of Traditional Chinese Medicine | 2021 | Guiding principles |
| 272 | Technical Guiding Principles for Pharmaceutical Change Research of Marketed Traditional Chinese Medicine (TCM) (Trial Implementation) | National Administration of Traditional Chinese Medicine | 2021 | Guiding principles |
| 273 | Announcement on Matters Concerning Encouraging Enterprises and Social Third Parties to Participate in the Formulation and Revision of Traditional Chinese Medicine (TCM) Standards | National Medical Products Administration | 2022 | Announcements |
| 274 | Announcement on Issuing the "Good Agricultural Practice (GAP) for Chinese Medicinal Materials" | National Medical Products Administration | 2022 | Announcements |
| 275 | Notice on Establishing the Expert Advisory Committee for Strategic Decision-Making on Traditional Chinese Medicine (TCM) Management | National Medical Products Administration | 2022 | Notifications |
| 276 | Announcement on Matters Concerning the Implementation of the "National Processing Standards for Traditional Chinese Medicine (TCM) Decoction Pieces" | National Medical Products Administration | 2022 | Announcements |
| 277 | Opinions on the "Special Provisions on Traditional Chinese Medicine (TCM) Registration Management (Draft for Comment)" | National Medical Products Administration | 2022 | Announcements |
| 278 | Opinions on the "Key Information Table of Ancient Classic Prescriptions (25 Prescriptions) (Draft for Comment)" | National Medical Products Administration, National Administration of Traditional Chinese Medicine | 2022 | Notifications |
| 279 | Announcement on Issuing the "Guiding Principles for Clinical R&D of New Traditional Chinese Medicine (TCM) Compound Preparations Based on Human Use Experience (Trial Implementation)" and "Guiding Principles for Communication Under the 'Three-Combination' Registration Evaluation Evidence System (Trial Implementation)" | National Medical Products Administration | 2022 | Announcements |
| 280 | Announcement on Issuing the "Guiding Principles for Writing Declaration Materials on Traditional Chinese Medicine (TCM) Theory of New TCM Compound Preparations (Trial Implementation)" and "Guiding Principles for Writing Instructions of TCM Compound Preparations of Ancient Classic Prescriptions (Trial Implementation)" | National Medical Products Administration | 2022 | Announcements |
| 281 | Notice on Issuing the "14th Five-Year Plan for the Development of Traditional Chinese Medicine (TCM) Talents" | National Administration of Traditional Chinese Medicine | 2022 | Notifications |
| 282 | Guiding Opinions on Medical Insurance Supporting the Inheritance and Innovative Development of Traditional Chinese Medicine (TCM) | National Healthcare Security Administration, National Administration of Traditional Chinese Medicine | 2022 | Opinions |
| 283 | Notice on the Progress of Traditional Chinese Medicine (TCM) Work in the Field of Maternal and Child Health | National Health Commission of China, National Administration of Traditional Chinese Medicine | 2022 | Announcements |
| 284 | Notice on Issuing the "Development Plan for Promoting the High-Quality Integration of Traditional Chinese Medicine (TCM) into the Joint Construction of the 'Belt and Road Initiative' (2021-2025)" | National Administration of Traditional Chinese Medicine | 2022 | Notifications |
| 285 | Announcement on Matters Concerning the Implementation of the "National Processing Standards for Traditional Chinese Medicine (TCM) Decoction Pieces" | State Food and Drug Administration | 2022 | Announcements |
| 286 | Notice on Issuing the "14th Five-Year Plan for the Informatization Development of Traditional Chinese Medicine (TCM)" | National Administration of Traditional Chinese Medicine | 2022 | Notifications |
| 287 | Notice on Issuing the "Guidelines for the Development of Forestry and Grassland Chinese Medicinal Materials Industry" | Office of the Forestry and Grassland Bureau | 2022 | Notifications |
| 288 | Notice on Issuing the "14th Five-Year Plan for the Development of Traditional Chinese Medicine (TCM)" | General Office of the State Council | 2022 | Notifications |
| 289 | Notice on Issuing the "14th Five-Year Action Plan for the Project to Improve the Service Capacity of Traditional Chinese Medicine (TCM) at the Grassroots Level" | Chinese Medicine Administration Bureau, National Health Commission, National Development and Reform Commission, Ministry of Education, Ministry of Finance, Ministry of Human Resources and Social Security, Ministry of Culture and Tourism, Medical Insurance Bureau, Drug Administration Bureau, Central Military Commission Logistics Support Department, Health Bureau | 2022 | Notifications |
| 290 | Notice on Issuing the "14th Five-Year Plan for the Development of Traditional Chinese Medicine (TCM)" | State Council | 2022 | Notifications |
| 291 | Opinions on Strengthening the Work of Traditional Chinese Medicine (TCM) Talents in the New Era | National Administration of Traditional Chinese Medicine, Ministry of Education, Ministry of Human Resources and Social Security, National Health Commission | 2022 | Opinions |
| 292 | Notice on Carrying Out the Special Activity of Traditional Chinese Medicine (TCM) Health Promotion in the Healthy China Initiative | Health China Action Promotion Office, Office of the National Health Commission, Office of the National Commission for Traditional Chinese Medicine | 2022 | Notifications |
| 293 | Implementation Regulations of the Drug Administration Law of the People's Republic of China (Draft for Comment) | State Council | 2022 | Ordinances |
| 294 | Guiding Principles for Writing Declaration Materials on Traditional Chinese Medicine (TCM) Theory of New TCM Compound Preparations (Trial Implementation) | National Administration of Traditional Chinese Medicine | 2022 | Guiding principles |
| 295 | Technical Guiding Principles for Sample Research for Toxicological Research of New Traditional Chinese Medicine (TCM) (Trial Implementation) | National Administration of Traditional Chinese Medicine | 2022 | Guiding principles |
| 296 | Guiding Principles for Clinical R&D of New Traditional Chinese Medicine (TCM) Compound Preparations Based on Human Use Experience (Trial Implementation) | National Administration of Traditional Chinese Medicine | 2022 | Guiding principles |
| 297 | Notice on Issuing Several Measures for Further Strengthening the Scientific Supervision of Traditional Chinese Medicine (TCM) and Promoting Its Inheritance and Innovative Development | State Council | 2023 | Notifications |
| 298 | Policy Interpretation of the "Special Provisions on Traditional Chinese Medicine (TCM) Registration Management" | National Administration of Traditional Chinese Medicine | 2023 | Rules and Requirements |
| 299 | Notice on Issuing the "Key Information Table of Ancient Classic Prescriptions (25 Prescriptions Including 'Zhuye Shigao Decoction')" | National Medical Products Administration, National Administration of Traditional Chinese Medicine | 2023 | Notifications |
| 300 | Notice on Issuing the "Key Information Table of Ancient Classic Prescriptions (7 Pediatric Prescriptions Including 'Yigong Powder')" | National Medical Products Administration, National Administration of Traditional Chinese Medicine | 2023 | Notifications |
| 301 | Notice on Establishing an Expert Working Group for Research on Regulatory Policies and Technical Requirements for Substitutes of Rare and Endangered Chinese Medicinal Materials | National Medical Products Administration | 2023 | Notifications |
| 302 | Issuance of the "Special Provisions on Traditional Chinese Medicine (TCM) Registration Management" | National Medical Products Administration | 2023 | Notifications |
| 303 | Notice on Establishing an Expert Working Group for Good Agricultural Practice (GAP) of Chinese Medicinal Materials | National Medical Products Administration | 2023 | Notifications |
| 304 | Regulations on the Protection of Traditional Chinese Medicine (TCM) Varieties | National Administration of Traditional Chinese Medicine | 2023 | Ordinances |
| 305 | Notice on Further Strengthening the Construction of Pediatrics in Traditional Chinese Medicine (TCM) Hospitals | National Administration of Traditional Chinese Medicine | 2023 | Notifications |
| 306 | Notice on Further Giving Play to the Characteristics and Advantages of Traditional Chinese Medicine (TCM) in the Medical Treatment of Novel Coronavirus Infection | National Administration of Traditional Chinese Medicine | 2023 | Notifications |
| 307 | Announcement on Issuing the "Special Provisions on Traditional Chinese Medicine (TCM) Registration Management" | State Food and Drug Administration | 2023 | Announcements |
| 308 | Issuance of the Implementation Plan for Major Projects for the Revitalization and Development of Traditional Chinese Medicine (TCM) | General Office of the State Council | 2023 | Notifications |
| 309 | Notice on Issuing the Implementation Plan for Major Projects for the Revitalization and Development of Traditional Chinese Medicine (TCM) | State Council | 2023 | Notifications |
| 310 | Notice on Issuing the "Implementation Plan for the '14th Five-Year Plan' Project for Promoting Traditional Chinese Medicine (TCM) Culture" | National Administration of Traditional Chinese Medicine, Central Propaganda Department, Ministry of Education, Ministry of Commerce, Ministry of Culture and Tourism, National Health Commission, State Administration of Radio, Film and Television, Cultural Relics Administration Department | 2023 | Notifications |
| 311 | Notice on Issuing the Standards for Traditional Chinese Medicine (TCM) Health Preservation and Health Care Services (Trial Implementation) | National Administration of Traditional Chinese Medicine | 2023 | Notifications |
| 312 | Notice on Issuing the "Measures for the Management of Mentorship Education for Traditional Chinese Medicine (TCM) Professional and Technical Personnel" | National Administration of Traditional Chinese Medicine | 2023 | Notifications |
| 313 | Issuance of the Drug Standard Management Measures, Clarifying the Formulation and Revision Procedures for Three Types of Standards | National Medical Products Administration | 2023 | Rules and Requirements |
| 314 | Announcement on Issuing the "Drug Standard Management Measures" | National Medical Products Administration | 2023 | Announcements |
| 315 | Announcement on Issuing the "Provisions on the Label Management of Traditional Chinese Medicine (TCM) Decoction Pieces" | National Medical Products Administration | 2023 | Rules and Requirements |
| 316 | Technical Guiding Principles for the Preparation Research of Drugs for Clinical Trials of New Traditional Chinese Medicine (TCM) (Trial Implementation) | National Administration of Traditional Chinese Medicine | 2023 | Guiding principles |
| 317 | Special Provisions on Traditional Chinese Medicine (TCM) Registration Management | National Administration of Traditional Chinese Medicine | 2023 | Rules and Requirements |
| 318 | Notice on Issuing Several Measures for Further Strengthening the Scientific Supervision of Traditional Chinese Medicine (TCM) and Promoting Its Inheritance and Innovative Development | National Medical Products Administration | 2023 | Notifications |
| 319 | Special Provisions on Traditional Chinese Medicine (TCM) Registration Management | National Administration of Traditional Chinese Medicine | 2023 | Rules and Requirements |
| 320 | Notice on Issuing Several Measures for Further Strengthening the Scientific Supervision of Traditional Chinese Medicine (TCM) and Promoting Its Inheritance and Innovative Development | National Medical Products Administration | 2023 | Notifications |
| 321 | Announcement on Issuing the "Special Provisions on Traditional Chinese Medicine (TCM) Registration Management" | National Medical Products Administration | 2023 | Announcements |
| 322 | Announcement on Issuing the "Drug Standard Management Measures" | National Medical Products Administration | 2023 | Announcements |
| 323 | Drug Standard Management Measures | National Medical Products Administration | 2023 | Methods |
| 324 | Announcement on the Issuance of Special Provisions for the Management of Traditional Chinese Medicine Standards | National Medical Products Administration | 2024 | Announcements |
| 325 | Announcement on Matters Concerning Supporting the Development of Substitutes for Rare and Endangered Chinese Medicinal Materials | National Medical Products Administration | 2024 | Announcements |
| 326 | Opinions on the "Special Provisions on the Supervision and Management of Traditional Chinese Medicine (TCM) Production (Draft for Comment)" | National Medical Products Administration | 2024 | Opinions |
| 327 | Notice on Publicly Soliciting Opinions on the "Key Information Table of Ancient Classic Prescriptions (Draft for Comment)" | National Administration of Traditional Chinese Medicine | 2024 | Notifications |
| 328 | Opinions on the "Special Provisions on Traditional Chinese Medicine (TCM) Standard Management (Draft for Comment)" | National Medical Products Administration | 2024 | Opinions |
| 329 | Announcement on Matters Concerning Supporting the Development of Substitutes for Rare and Endangered Chinese Medicinal Materials | National Medical Products Administration, National Administration of Traditional Chinese Medicine | 2024 | Announcements |
| 330 | Establishment and Implementation of the Drug Traceability System; Implementation of the "Measures for the Supervision and Management of Drug Distribution and Use Quality" | National Medical Products Administration | 2024 | Announcements |
| 331 | Opinions on Accelerating the Construction of Advantageous Specialties of Traditional Chinese Medicine (TCM) | National Administration of Traditional Chinese Medicine | 2024 | Opinions |
| 332 | Notice on Issuing the "Standards for Information and Digital Construction of Traditional Chinese Medicine (TCM) Hospitals (2024 Edition)" | National Administration of Traditional Chinese Medicine | 2024 | Notifications |
| 333 | Notice on Issuing the "Key Work Tasks for Deepening the Reform of the Medical and Health System in 2024" | State Council | 2024 | Notifications |
| 334 | Announcement on Issuing the "Special Provisions on Traditional Chinese Medicine (TCM) Standard Management" | National Medical Products Administration | 2024 | Announcements |
| 335 | Announcement on Issuing the "Measures for the Management of Regional Folk Customary Medicinal Materials" | National Medical Products Administration, National Administration of Traditional Chinese Medicine | 2024 | Announcements |
| 336 | Notice on Issuing the "Several Opinions on Promoting the Development of Digital Traditional Chinese Medicine (TCM)" | National Administration of Traditional Chinese Medicine, National Data Bureau | 2024 | Notifications |
| 337 | Notice on Issuing the "Traditional Chinese Medicine (TCM) Standardization Action Plan (2024-2026)" | National Administration of Traditional Chinese Medicine | 2024 | Notifications |
| 338 | Announcement on Issuing the "Special Provisions on Traditional Chinese Medicine (TCM) Standard Management" | National Medical Products Administration | 2024 | Announcements |
| 339 | Notice on Issuing the Management Measures and Construction Standards for the Inheritance Bases of Traditional Chinese Medicine (TCM) Processing Technology | National Administration of Traditional Chinese Medicine | 2024 | Notifications |
| 340 | Technical Guiding Principles for the Research on Characteristic Spectra of Traditional Chinese Medicine (TCM) Preparations (Trial Implementation) | National Administration of Traditional Chinese Medicine | 2024 | Guiding principles |
| 341 | Technical Guiding Principles for the Stability Research of Traditional Chinese Medicine (TCM) Preparations (Trial Implementation) | National Administration of Traditional Chinese Medicine | 2024 | Guiding principles |
| 342 | Traditional Chinese Medicine (TCM) Standardization Action Plan (2024-2026) | National Administration of Traditional Chinese Medicine | 2024 | Guiding principles |
| 343 | Special Provisions on Traditional Chinese Medicine (TCM) Standard Management | National Administration of Traditional Chinese Medicine | 2024 | Rules and Requirements |
| 344 | Opinions on Comprehensively Deepening the Reform of Drug and Medical Device Supervision | State Council | 2024 | Guiding principles |
| 345 | Announcement on Issuing the "Special Provisions on Traditional Chinese Medicine (TCM) Standard Management" | National Medical Products Administration | 2024 | Announcements |
| 346 | Notice on Issuing the "Traditional Chinese Medicine (TCM) Standardization Action Plan (2024-2026)" | National Administration of Traditional Chinese Medicine | 2024 | Notifications |
| 347 | Key Work Tasks for Deepening the Reform of the Medical and Health System in 2024 | State Council | 2024 | Guiding principles |
